# Aminosilane Coupling Agents at the Boron Carbide/Poly(Vinyl Chloride) Interface: Substitution, Minor Dehydrochlorination and C–Cl Bond Weakening

**DOI:** 10.3390/nano16201316

**Published:** 2026-10-18

**Authors:** Yetkin Senemoğlu

**Affiliations:** Department of Chemistry and Chemical Processing Technologies, Çaycuma Food and Agriculture Vocational School, Zonguldak Bülent Ecevit University, 67900 Zonguldak, Türkiye; yetkin.senemoglu@beun.edu.tr

**Keywords:** neutron shielding, boron carbide, poly(vinyl chloride), nanocomposite interface, silane coupling agent, dehydrochlorination, nucleophilic substitution, radiolysis, density functional theory, transition state theory

## Abstract

Boron carbide in a hydrogenous polymer is a standard thermal-neutron shield. Under a preliminary cross-section estimate, poly(vinyl chloride) (PVC) reaches the absorption of high-density polyethylene carrying 10 wt% $B_{4}C$ with 6.2 wt%, and so needs less filler and less coupling agent. Whether an aminosilane coupling agent is inert toward such a matrix was examined computationally. Substitution and HCl elimination by the pendant amine at a backbone C–Cl unit were located as verified transition states for free and grafted models, sampled conformationally and compared over a range of dielectric environments. The semiempirical GFN2-xTB and GFN1-xTB methods favor elimination through the zero-point advantage of a synchronous proton transfer. Dispersion-corrected hybrid density functional theory (DFT) does not: its elimination transition state is asynchronous, the zero-point advantage falls from 16 to 7 kJ mol^−1^, and substitution is favored by 12 to 19 kJ mol^−1^ (single transition-state conformers, mixed DFT/GFN2-xTB Hessians). The semiempirical prediction of dominant elimination is therefore unreliable; the coupling agent most likely reacts with PVC mainly by *N*-alkylation, although no product ratio for a real composite follows. At the DFT level, the half-life of a grafted amine at 453 K is on the order of hours to days, an order-of-magnitude estimate for an idealized bimolecular encounter. The minor elimination channel leaves allylic C–Cl bonds 76.5 kJ mol^−1^ weaker than the backbone. For PVC-matrix shields, non-basic methacryloxypropyl and glycidoxypropyl silanes, which carry no nucleophilic nitrogen, are the safer choice where amine-induced HCl loss and ammonium chloride formation at the interface are to be avoided, as far as the amine-mediated pathways studied here are concerned.

## 1. Introduction

Shielding against thermal neutrons requires two functions in one material: a hydrogenous medium to moderate the incident flux, and a nuclide with a large absorption cross section to capture the thermalized neutrons. Boron carbide ($B_{4}C$) supplies the second function unusually well, since B10 has one of the largest thermal-neutron capture cross sections of any stable nuclide, and a polymer supplies the first. Composites built on this principle have been characterized in high-density polyethylene (HDPE) [1,2], low-density polyethylene (LDPE) [3], polyimide [4], epoxy [5,6,7] and several elastomers [8,9]. Poly(vinyl chloride) (PVC) is an established shielding matrix in its own right, but the published work on it is confined to photon attenuation. PVC composites filled with bismuth oxide [10], with lead oxide [11], with mixed lead and tungsten oxides [12] and with tungsten oxide [13] have all been developed and characterized as flexible, formable gamma or X-ray shielding. In every case the filler is a high-*Z* photon absorber and the matrix is chosen for processability.

What does not appear in this literature is PVC carrying a *neutron* absorber. A search of Scopus and Web of Science covering 1990 to 2026, combining the matrix terms “poly(vinyl chloride)” and “PVC” with “boron carbide”, “$B_{4}C$”, “boron”, “neutron shielding” and “thermal neutron”, returned photon-shielding composites and boron-filled polyolefins but no boron carbide-filled PVC characterized for neutron attenuation; to the best of the author’s knowledge, such a composite has not been reported.

The combination is not arbitrary, and Section 3.2 quantifies why. PVC is the weaker moderator, holding about half the hydrogen of polyethylene per unit volume, but its chlorine is itself a thermal absorber, and the two effects do not cancel: an unfilled PVC captures thermal neutrons seventeen times as strongly as unfilled polyethylene, so less boron carbide is needed to reach a given capture. Since every particle of filler brings interfacial area, and every unit of interfacial area brings coupling agent, that difference matters for the chemistry as much as for the shielding.

The obstacle is the interface, and it is a nanoscale one. Whatever the size of the filler particle, the region that transfers load and that the coupling agent occupies is a grafted molecular layer one to two nanometers thick, and its chemistry is not that of either bulk phase. Reducing the filler to submicron or nanoscale powder, which is what improves dispersion and attenuates the self-shielding of individual particles, multiplies the specific surface area and therefore multiplies that interphase per unit volume of composite. A hard ceramic particle dispersed in a compliant polymer transfers load and resists debonding only as well as that layer allows, and untreated $B_{4}C$ disperses poorly. The standard remedy is a silane coupling agent, and for boron carbide this has direct precedent: silane surface modification measurably improves $B_{4}C$ adhesion to epoxy [14], and the closest analog to the present system in the shielding literature is a silane-modified boric acid/HDPE composite developed specifically for radiation shielding [15]. Among available silanes, 3-aminopropyltriethoxysilane (APTES) is used routinely, including on $B_{4}C$ in polyimide shielding composites [4], and its surface chemistry on oxides is well characterized: the ethoxy groups hydrolyze to silanols which condense with surface hydroxyls to give mono-, bi- or tridentate attachment, with the speciation set by water content, temperature and hydroxyl density [16,17,18,19,20]. Its selection for a given matrix is rarely argued in the composite literature, which is the habit this paper examines.

Silanes are selected from tables organized by which resin the organofunctional group is meant to react with. That logic works for a thermoset, where the intended reaction is the only one available: an amine terminus is ideal for epoxy because the amine and the epoxide are the reactive pair. PVC sits awkwardly in such tables because it is not expected to react with anything during compounding, so the coupling agent is chosen for polarity and plasticizer compatibility, and the amine is treated as a polar head group. The possibility that it might act as a Brønsted base toward the polymer backbone does not enter the analysis.

It should, because PVC degrades by dehydrochlorination: loss of HCl generates an allylic chloride whose subsequent elimination is far easier than the first, producing a conjugated polyene sequence that discolors and embrittles the material [21,22,23]. The controlling structures are the labile chlorines, allylic and tertiary, whose formation and disproportionate influence on stability were established by Hjertberg and Sörvik [24,25] and whose mechanistic role has been reviewed [26] and studied computationally [27] since. The barriers are known: thermogravimetry gives an apparent activation energy of 115.7 ± 0.7 kJ mol^−1^ for the dehydrochlorination step of PVC pyrolysis, against a computed 135 kJ mol^−1^ for the HCl-catalyzed path [28], and a model-free analysis of chlorinated PVC gives 140 kJ mol^−1^ for the same stage [29]. Primary amines are participants in this chemistry rather than spectators. X-ray photoelectron spectroscopy (XPS) of PVC treated with ethylenediamine shows elimination and substitution occurring in competition, with both C–N bond formation and polyene formation detectable in the product [30]; the chemical modification of PVC by nucleophiles has been reviewed [31], and the fine balance between the two channels has been revisited recently in the context of chemical recycling [32].

For a shielding composite this matters twice over, and the second time is specific to the service environment. A shielding material exists to sit in a radiation field, and a PVC matrix in that field undergoes radiolytic dehydrochlorination throughout its service life. The radiation chemistry is well established: gas evolution and property loss in irradiated rigid PVC films [33], the dependence of HCl yield on humidity and temperature under gamma irradiation [34], dose-rate effects on the thermal stability of irradiated formulations [35], the comparison of electron and gamma irradiation [36], radiolytic degradation and its stabilization [37,38], and the radical populations detectable by electron spin resonance (ESR) after irradiation [39,40]. Filled polymers age under irradiation in ways that depend on the filler and its surface treatment [41,42,43], and a $B_{4}C$/epoxy shield has been shown to change measurably after neutron irradiation [5].

The connection examined here is as follows. If a grafted aminosilane chemically dehydrochlorinates the PVC it is supposed to couple to, the product of that reaction is an allylic chloride, and an allylic C–Cl bond is weaker than a backbone one, which makes it a candidate for preferential cleavage under irradiation. The coupling agent would then not merely damage the matrix during compounding; it could also create, at the interface and in proportion to the grafting density, weak-bond defects on which radiolytic degradation may act preferentially, and both effects would scale with the interfacial area that a finer filler is chosen to provide. The competing channel, substitution, would consume the same C–Cl units and the amine itself, converting the coupling agent into a polymer-bound alkylammonium chloride; which of the two channels dominates is therefore the first question this work answers.

This paper tests that proposition computationally. The specific questions are: what the composite is worth as a shield, and therefore how much coupling agent it needs; whether grafting is thermodynamically accessible; whether the pendant amine prefers substitution or elimination when it meets a C–Cl unit, how that preference depends on the grafting state and on the attachment mode, and above all what decides it; whether the elimination product is autocatalytically activated and carries a weaker C–Cl bond; and whether the behavior across a series of organofunctional silanes can be ranked from a simple descriptor, which would turn a negative finding into a formulation rule.

Two methodological commitments follow from the size of the effects being chased. Every barrier reported here belongs to a transition state that was optimized without constraints and verified by its vibrational spectrum, because a maximum along a constrained scan is not a saddle point and cannot be treated as one. At the density functional level this verification uses the curvature in a subspace spanned by the softest modes and the semiempirical reaction mode, and Section 3.7 shows that the density functional substitution structure on the larger diborate cluster model is only an approximate saddle point. And every channel comparison is made between free energies of two transition states of identical composition, because the two channels proceed from rapidly interconverting encounter complexes and the Curtin–Hammett principle then makes the difference of transition-state free energies the only quantity that governs the branching [44]. Section 3.5 shows that both commitments change the electronic comparison substantially, and in two pairs its sign. A third commitment is that the semiempirical results are checked against dispersion-corrected density functional theory (DFT), and Section 3.6 shows that this check changes the answer most of all.

## 2. Materials and Methods

### 2.1. Computational Strategy and Level of Theory

This is a computational study; no new experimental data were produced, and every experimental value quoted in the tables and figures is a literature value whose source is cited where it appears. Conformational space was sampled with distance-geometry embedding and a classical force field used as a geometry filter: force-field energies decide which conformers are carried forward (Section 2.2) but enter no reported quantity. Every surviving conformer was re-optimized with the GFN2-xTB semiempirical tight-binding method (geometries, frequencies and noncovalent interactions extended tight binding) [45] as implemented in the xtb program version 6.7.1 [46]. Transition-state searches and vibrational analyses used the same Hamiltonian through the tblite library, which reproduces the xtb binary’s GFN2-xTB energies with the analytical linearized Poisson–Boltzmann (ALPB) solvation model [47] to better than 10^−5^ hartree on these systems; that agreement was checked on every structure carried between the two programs. GFN2-xTB employs anisotropic second-order density fluctuations, multipole electrostatics and a density-dependent dispersion treatment, and is parameterized across the periodic table, which makes it suited to a study that explores many structures rather than a few.

Because the selectivity reported here rests on free-energy differences of a few to a few tens of kJ mol^−1^, which is the order of the typical error of a semiempirical Hamiltonian, the semiempirical results were checked with dispersion-corrected hybrid density functional theory at two levels. First, single-point energies were computed at every GFN2-xTB saddle point of the eight amine/substrate pairs with PBE0 [48] and the D3 dispersion correction with Becke–Johnson damping [49,50] in the def2-SVP basis [51], using PySCF [52] with density fitting, in the gas phase and in the integral-equation-formalism polarizable continuum model (IEF-PCM) [53] at a relative permittivity of 3.4, and for three pairs (*n*-propylamine and the monodentate graft on the single boron site and on the diborate) also at 7.4. Second, for the two systems on which the conclusions chiefly rest, the saturated PVC model with *n*-propylamine and with the monodentate grafted silane, both transition states and the separated reactants were re-optimized at PBE0-D3(BJ)/def2-SVP/IEF-PCM(*ε* = 3.4) with the same saddle-point optimizer used for the semiempirical searches (Section 2.4), started from the GFN2-xTB saddle point with the GFN2-xTB Hessian as the initial Hessian, to a maximum force of 0.025 eV Å^−1^. The substitution saddle point of the grafted silane cycled on the rotation of peripheral hydroxyl groups and was taken at a maximum force of 0.035 eV Å^−1^, the residual force lying on hydroxyl atoms away from the reacting core (Table S7). The diborate pair of Section 2.9 was re-optimized in the same way; its elimination saddle point was taken at a maximum force of 0.046 eV Å^−1^, and its substitution search, which did not converge, at the point of lowest residual force, 0.17 eV Å^−1^ (Section 3.7, Table S7); that structure is an approximate saddle point, not a converged transition state, and is used only qualitatively. The optimizations used an integration grid of level 2 and a Lebedev order of 17 for the cavity surface, and the final energies were recomputed with a level-3 grid and a Lebedev order of 29. The analytic Hessian with the continuum exceeded the available memory, and a Hessian without the continuum at the continuum-optimized geometry is not a Hessian of the surface on which the saddle point was found, so both were replaced by quantities evaluated on the continuum surface itself. Each saddle point was verified by the curvature of that surface in the subspace of nine mass-weighted directions, the eight softest modes of the GFN2-xTB Hessian at the DFT geometry and the GFN2-xTB reaction mode, from central differences of analytic PBE0-D3(BJ)/IEF-PCM gradients; the projected Hessian has exactly one negative eigenvalue for each of these six structures, including the approximate diborate substitution saddle point. For substitution its eigenvector lies mainly on the reacting core; for the asynchronous elimination saddle points of Section 3.6 and Section 3.7 it is the departure of the chloride coupled to the motion of the transferred proton, the amino group and the two carbons, which together carry about three quarters of its amplitude. The 150 cm^−1^ threshold of Section 2.4 was set for the semiempirical searches; at the DFT level the reaction mode of these asynchronous elimination saddle points is the soft departure of the chloride (−110 to −128 cm^−1^), and they were accepted on the single negative curvature and on this composition of the mode. An intrinsic reaction coordinate calculation with the continuum was beyond the available memory. The four saddle points of the two principal pairs were instead connected to their two sides by downhill relaxations on the same surface: each was displaced in both directions along the reaction mode of its mixed Hessian (described below), by 0.10 Å for the atom that moves most, and relaxed at PBE0-D3(BJ)/def2-SVP/IEF-PCM (*ε* = 3.4) with a quasi-Newton optimizer whose initial Hessian was that mixed Hessian with its eigenvalues taken by magnitude, until the bonds formed and broken had identified one side of the reaction and the maximum force had fallen below 0.5 eV Å^−1^; the two slower substitution relaxations of the grafted silane were instead stopped once the assignment had held for three consecutive steps. Section 3.6 describes the outcome, and Table S11 lists the end points. The thermal term was obtained from a Hessian whose rows and columns for the reacting core (the nitrogen and its hydrogens, the transferring β-hydrogen and its carbon, the chlorine-bearing carbon and its hydrogen, and the chlorine) were computed by forward differences of the same DFT gradients, with the remaining block taken from GFN2-xTB at the same geometry, and treated with the quasi-rigid-rotor/harmonic-oscillator (quasi-RRHO) scheme of Section 2.5. The separated reactants were optimized on the same surface to a maximum force of 0.05 eV Å^−1^, at which their energies no longer changed by more than 0.01 kJ mol^−1^, and their thermal terms were obtained from mixed Hessians with the same atoms in the DFT block as in the transition states, so that the GFN2-xTB part cancels in the barrier as far as it can. Energies were checked with the def2-TZVP basis set and with the B3LYP-D3(BJ) and M06-2X-D3(0) functionals, as single points at the PBE0-D3(BJ)/def2-SVP geometries.

### 2.2. Structure Generation

Starting geometries were generated from simplified molecular-input line-entry system (SMILES) strings with explicit stereochemistry using RDKit and the experimental-torsion knowledge distance geometry (ETKDG) algorithm in its third version (ETKDGv3) [54], with a fixed random seed (20260831) and an embedding-time root-mean-square deviation (RMSD) pruning threshold of 0.6 Å. Up to 150 conformers were embedded per species and pre-optimized with the Merck molecular force field MMFF94s, with a universal force field (UFF) fallback for species outside MMFF’s parameterization (boron-containing models and HCl); the ten lowest in force-field energy were retained for quantum-chemical refinement (twelve for the three hexamers and the tetramer, for which two further force-field conformers were also refined; neither became the GFN2-xTB minimum). The 150-conformer pool is an upper limit on the embedding, not a target: after RMSD pruning the small species yield fewer than ten distinct conformers, in which case all were kept. For the flexible species the kept conformers span 1.9 to 31.4 kJ mol^−1^ in force-field energy, and the first discarded conformer lies 2.3 to 26.2 kJ mol^−1^ above the force-field minimum; the energy windows of every species are listed in Table S1. Since the force field serves only to remove clashed or unphysical embeddings, the relevant question is whether the GFN2-xTB ordering of the kept set is stable, and it is: the GFN2-xTB energy range of the refined set is 1.7 to 24.5 kJ mol^−1^ (Section 3.3). Structure handling used the Atomic Simulation Environment [55].

The PVC models comprise three atactic hexamer stereosequences (*RSSRSR*, *RRSSRS*, *RSRSRS*), an oligomer series at *n* = 2, 4 and 6, the dyad model compound 2,4-dichloropentane, and 4-chloro-2-pentene as the allylic chloride formed after one dehydrochlorination event. The silane models comprise the parent APTES, its mono- and dihydrolyzed forms, 3-aminopropylsilanetriol, and three grafted species differing in attachment mode: $H_{2}N(CH_{2}{)}_{3}Si(OH{)}_{2}-O-B(OH{)}_{2}$ (monodentate, $T^{1}$, two silanols left free), $H_{2}N(CH_{2}{)}_{3}Si(OH)[O-B(OH{)}_{2}{]}_{2}$ (bidentate, $T^{2}$) and $H_{2}N(CH_{2}{)}_{3}Si[O-B(OH{)}_{2}{]}_{3}$ (tridentate, $T^{3}$). All three are examined because the speciation on real oxide surfaces favors the higher modes [16] and the silanols left free after grafting can hydrogen-bond to the leaving chloride. Boric acid serves as the molecular analog of a hydroxylated surface boron site; Section 3.1 tests that choice against a periodic surface, and Section 2.9 describes the larger cluster models used to test its energetics. *n*-Propylamine models the aminopropyl terminus stripped of its silanol head.

### 2.3. Conformer Refinement and Thermochemistry of Minima

Refinement used two passes. Every force-field conformer was optimized with tight convergence and the set re-ranked at the GFN2-xTB level; the global minimum of each species was then subjected to a combined optimization and numerical Hessian calculation, giving harmonic frequencies and the thermostatistics described in Section 2.5. All sixteen closed-shell species listed in Section 3.3 were confirmed as true minima, with no vibrational mode below −10 cm^−1^ after removal of the translational and rotational modes.

### 2.4. Locating and Verifying Transition States

Reaction paths were first mapped by a concerted two-coordinate relaxed scan, and that scan is used here only to generate a starting guess. Encounter complexes were assembled deterministically: for substitution the base atom was placed on the backside of the C–Cl bond, collinear with the Cl–Cα axis; for elimination it was placed along the Cβ–H vector of the β-hydrogen most nearly anti-periplanar to the leaving C–Cl bond. The silane was rigidly rotated about the approach axis through 72 increments and the orientation maximizing the shortest inter-fragment contact retained, with the approach distance increased in 0.5 Å steps until that contact reached 1.8 Å, since a bulky graft cannot be placed at the same distance as a small amine without atoms overlapping. The scan then drove *d*(N···Cα) from 3.30 to 1.55 Å for substitution, or *d*(N···Hβ) from 2.60 to 1.05 Å for elimination, together with *d*(Cα–Cl) from its equilibrium value to 3.20 Å, in 26 steps with a constraint force constant of 2.0 a.u.

Each scan maximum was then refined to a genuine first-order saddle point on the unconstrained surface with the Sella partitioned rational-function optimizer [56,57], to a maximum force of 0.02 eV Å^−1^. The search was seeded with a Cartesian direction built from the intended reaction coordinate (the base closing on Cα and the chloride leaving, or the β-hydrogen moving to the base and the chloride leaving), so that it follows the intended mode rather than whichever mode happens to be softest at the guess. A full harmonic Hessian was then computed by finite differences of analytical gradients.

A structure was accepted as a transition state only if it satisfied three conditions: exactly one imaginary mode, a mode being counted as imaginary when its frequency lies below −10 cm^−1^ (the numerical noise level of a finite-difference Hessian); that mode larger than 150 cm^−1^ in magnitude, so that it is a bond-breaking motion and not a floppy torsion; and an overlap above 0.25 between that mode and the seeding direction, so that it belongs to the reaction being studied. Where the search returned a second imaginary mode below 60 cm^−1^ in magnitude, that mode is a hindered rotor of a pendant hydroxyl rather than a second reaction coordinate; such structures are used, flagged in the tables, and their soft mode is treated as a low-frequency vibration in the quasi-RRHO treatment of Section 2.5. Anything else was rejected and reported as a failure. Searches that failed the test were retried, in this order, in internal coordinates, from a geometry displaced along the seeding direction, from a geometry displaced along a second imaginary mode, and with an exact initial Hessian in Cartesian and then in internal coordinates. The retries in internal coordinates and with an exact initial Hessian are not seeded, and for them the overlap test above decides whether the saddle point belongs to the intended reaction. Of the 42 searches, 5 ended at the first, seeded attempt, 28 at the retry in internal coordinates, 8 with an exact initial Hessian in Cartesian coordinates and 1 with an exact initial Hessian in internal coordinates (Table S2).

*Conformational sampling of the transition states.* A single encounter complex per channel samples one arrangement of the flexible silane and polymer chains out of many. Every transition state of the eight amine/substrate pairs was therefore sampled further. The reacting core (the base nitrogen, the atom it attacks, Cα, the leaving chlorine and, for elimination, Cβ) was held at its saddle-point position while the rest of the complex was propagated by Langevin molecular dynamics with GFN2-xTB/ALPB for 2.4 ps; the elevated temperature of 800 K serves only to carry the silanol, alkyl and backbone torsions across their barriers within that time, and no quantity is computed at it. Ten snapshots, one every 240 fs, were relaxed with the core still fixed and then released and optimized as first-order saddle points with the same optimizer and the same acceptance test as above; duplicates (energies within 0.1 kJ mol^−1^ and heavy-atom RMSD below 0.1 Å) were merged. This follows the practice of multi-conformer transition-state theory [58]. The number of starting structures, converged searches and distinct transition-state conformers for every reaction is given in Table S2.

### 2.5. Barrier Differences, Free Energies and Tunneling

The two channels, bimolecular nucleophilic substitution ($S_{N}2$) and bimolecular elimination (E2), start from two different encounter complexes, assembled differently and relaxed under different constraints; their energies differ by up to 10.5 kJ mol^−1^. Measuring each barrier from its own complex and then subtracting is therefore not a well-defined comparison, and it is not the comparison that governs the branching. Two weakly bound complexes of the same pair of molecules interconvert far faster than either reacts, so by the Curtin–Hammett principle [44] the product ratio depends on the difference of the two transition-state free energies and on nothing else. That difference needs no reference state at all, because the two transition states of a pair have identical composition and their absolute energies are already commensurate:

(1) $$\Delta \Delta X^{‡}\;=\;X\left(TS,S_{N}2\right)\;-\;X\left(TS,E2\right),\;X\;=\;E,\;H\;or\;G$$

(2) $$\frac{k\left(E2\right)}{k\left(S_{N}2\right)}=\frac{\kappa_{E2}}{\kappa_{S_{N}2}}exp\left[\frac{\Delta \Delta G^{‡}}{RT}\right]$$

A positive $\Delta \Delta X^{‡}$ therefore means elimination is preferred. Where several conformers of a transition state were located, each channel enters Equation (2) through its conformer-summed rate, $\sum_{i}\kappa_{i}exp\left(-G_{i}/RT\right)$, which is the multi-conformer form of the same expression [58].

Enthalpies and entropies come from the same Hessians at 453.15 K, the middle of the PVC melt-processing window. Translational and rotational contributions are computed explicitly from the actual masses and inertia tensors rather than assumed to cancel; for two transition states of identical composition the translational terms cancel exactly and the rotational terms differ by at most 1.5 kJ mol^−1^. Every one of the 3*N* − 7 vibrations of a transition state enters the sums, the reaction coordinate being the only mode removed, so that the two transition states of a pair always contribute the same number of modes. Low-frequency modes are the weak point of any harmonic free energy, because the harmonic entropy diverges as a frequency goes to zero and these complexes carry several soft inter-fragment modes. The primary treatment is therefore Grimme’s quasi-RRHO interpolation [59], in which each mode is weighted between a harmonic oscillator and a free rotor with a switching frequency of 100 cm^−1^ and an averaging moment of inertia of 10^−44^ kg m^2^. As a sensitivity test the free energies were also computed with hard floors, raising every real mode below a threshold to that threshold [60], at 0 (the pure harmonic limit), 25, 50, 100 and 150 cm^−1^. Section 3.8 reports the result under all six treatments.

The imaginary frequencies differ greatly between the channels: under GFN2-xTB, about 960–1260 cm^−1^ for elimination, where a hydrogen is in flight, against 410–540 cm^−1^ for substitution. Tunneling is therefore a further asymmetry between them, and it is included through the Wigner correction $\kappa =1+\frac{1}{24}{\left(\frac{h\nu^{‡}}{k_{B}T}\right)}^{2}$ [61]. This is the crudest useful estimate and is reported as a lower bound on the effect.

Absolute barriers are measured from the separated reactants and converted from the 1 bar ideal-gas standard state to 1 mol L^−1^, which lowers a bimolecular activation free energy by $RTln\left(c^{\circ }RT/p^{\circ }\right)$ = 13.7 kJ mol^−1^ at 453 K. The pseudo-first-order rate constant of a grafted amine is then the bimolecular rate constant multiplied by the concentration of C–Cl units in the matrix, 22.4 mol L^−1^ for PVC of density 1.40 g cm^−3^, and its half-life is compared in Section 3.6 with the time a PVC compound spends in the melt during processing, of the order of minutes to tens of minutes, as in roll milling at 170 °C for up to 20 min [62].

### 2.6. Dielectric Environment

Both reactions generate an alkylammonium chloride ion pair. In vacuum such charge separation is strongly destabilized and the profile is dominated by electrostatics rather than by the chemistry of interest, so all semiempirical optimizations were run with ALPB [47] in chloroform. The permittivity of the medium in which the reaction occurs is not a single number. Rigid PVC at room temperature has a relative permittivity of about 3; above its glass transition the orientational α relaxation of the C–Cl dipoles contributes to the static permittivity, so the melt at 453 K responds more strongly, and the local environment of an amine at a filler interphase, among silanols and hydroxylated boron sites, is more polar again. The electronic term was therefore not evaluated at one assumed permittivity. The transition-state energies of every pair were re-evaluated at their optimized geometries in all twelve solvents for which ALPB is parameterized in the range *ε* = 2.4 to 78 (toluene 2.4, carbon disulfide 2.6, furan 2.9, diethyl ether 4.3, chloroform 4.7, ethyl acetate 6.0, tetrahydrofuran 7.4, dichloromethane 8.9, acetone 20.5, acetonitrile 36.6, dimethyl sulfoxide 46.7 and water 78.4) and in vacuum. Because each ALPB solvent has its own parameter set, the result is not a smooth function of *ε* and no interpolation between solvents is meaningful. The results are instead reported as a band: the median and range over the six solvents with 2.9 ≤ *ε* ≤ 8.9, which brackets glassy PVC, the melt and a moderately polar interphase. The low-permittivity solvents are reported separately, because the selectivity changes there. Independently of ALPB, the PBE0-D3(BJ) single points of Section 2.1 use IEF-PCM with the permittivity as a direct input, at 3.4 for every pair and at 7.4 for three of them. The zero-point and entropic terms are computed at the chloroform geometries and carried across unchanged. This assumes that the vibrational terms depend much less on the dielectric than the electronic term does; the assumption is not tested here and is stated among the limitations in Section 4.5.

### 2.7. Silane Series and the Basicity Descriptor

Eight organofunctional silanes were examined in their grafted form $R-Si(OH{)}_{2}-O-B(OH{)}_{2}$: aminopropyl, *N*-methylaminopropyl, *N*,*N*-dimethylaminopropyl, acetamidopropyl, glycidoxypropyl, methacryloxypropyl, vinyl and *n*-propyl. For each, the site that is protonated for the descriptor and the site that acts as the base in the reaction path were identified by the same substructure query, so that the two cannot disagree. An amide protonates on the carbonyl oxygen rather than on nitrogen, and using the nitrogen merely because it is the only one present would make the correlation meaningless. A relative proton affinity referenced to the aminopropyl case was used as the descriptor, so that the arbitrary absolute proton energy cancels:

(3) $$\delta PA\left(R\right)\;=\;\left[E\left(R\right)\;-\;E\left({RH}^{+}\right)\right]\;-\;\left[E\left(ref\right)\;-\;E\left({refH}^{+}\right)\right]$$

Both the neutral and the protonated form of every silane were taken as the lowest of an ensemble of 30 embedded and GFN2-xTB/ALPB-optimized conformers, because a protonated site can form an intramolecular hydrogen bond to a silanol or boron hydroxyl, and a single conformer of either form can include or miss that bond by chance. For each silane with a basic site, the maximum of the elimination scan against the PVC dyad was refined to a verified first-order saddle point with the protocol of Section 2.4, and the encounter complex from which the scan started was relaxed without constraints to its minimum; the elimination barrier is reported as the energy and the quasi-RRHO free energy of the transition state relative to that complex. The uncertainty of the barrier-descriptor regression was estimated by 10,000 bootstrap resamples and by leave-one-out refits. Vinyl and *n*-propyl carry no lone pair; protonation would give a carbocation, which is not a Brønsted basicity in any useful sense, so no descriptor is reported for them and no reaction path was run.

### 2.8. Bond Dissociation Energies

The primary radiolytic event in a chloropolymer is homolysis of a C–Cl bond to give a chlorine atom and a macroradical. Homolytic bond dissociation energies (BDEs) were computed as:

(4) $$BDE\;=\;E\left(R•\right)\;+\;E\left(Cl•\right)\;-\;E\left(R-Cl\right)$$

for the saturated PVC dyad, the allylic chloride produced by one elimination, and a tertiary chloride representing the other classic labile-chlorine defect. Radicals were treated as open-shell doublets. These calculations were run in the gas phase: homolysis produces neutral radicals, so there is no charge separation for a continuum to stabilize. A composition check enforces that the radical differs from R–Cl by exactly one chlorine atom, since a mistyped radical structure otherwise yields a nonsensical value silently. The computed values are electronic energy differences without zero-point or thermal corrections, compared below with measured bond dissociation enthalpies at 298 K; these corrections are expected to affect differences between chlorides much less than absolute values.

The PVC models were computed directly with PBE0-D3(BJ)/def2-SVP. Four small chlorides with measured bond dissociation enthalpies were run through the identical protocol as a validation set. Their experimental values were assembled as $BDE=\Delta_{f}H\left(R•\right)+\Delta_{f}H\left(Cl•\right)-\Delta_{f}H\left(R-Cl\right)$ from gas-phase enthalpies of formation in the National Institute of Standards and Technology (NIST) Chemistry WebBook [63], with $\Delta_{f}H(Cl•)$ = 121.30 kJ mol^−1^ [64].

### 2.9. Periodic Boron Carbide, the Surface Model and Larger Interface Models

The molecular models above stand in for a solid, so the solid was built and checked. Two ordered cells were constructed in the hexagonal setting of $R\bar{3}m$ (No. 166) with *a* = 5.5974 Å and *c* = 12.0715 Å from four Wyckoff orbits: two 18*h* orbits for the equatorial and polar icosahedral vertices, one 6*c* orbit for the chain terminus and one 3*b* site at (0, 0, ½) for the chain center. The starting free parameters were taken from the Rietveld refinement of Kolel-Veetil et al. [65] and cross-checked against the independent single-crystal refinement of Sologub et al. [66]. The two parameter sets are given in different origins and different orbit representatives, so they were compared by expanding both in the same cell and comparing the resulting bond-length distributions: the multiplicities are identical (9, 18, 18, 36 and 18 B–B contacts, 18 icosahedron-to-chain B–C contacts and 6 chain C–B contacts) and every corresponding distance agrees to within 0.02 Å, which establishes that the two describe the same structure.

The first cell is $B_{13}C_{2}$: a $B_{12}$ icosahedron with a C–B–C chain, carbon on the chain ends only, which is ordered in $R\bar{3}m$. The second is $B_{4}C$, obtained by replacing one polar icosahedral boron per icosahedron with carbon to give $B_{11}C(CBC)$, the arrangement identified as lowest in energy from first-principles nuclear magnetic resonance (NMR) analysis [67]. That substitution necessarily lowers the symmetry below $R\bar{3}m$, which is why no ordered $R\bar{3}m$ cell of $B_{4}C$ stoichiometry exists and why the extent of genuine carbon disorder remains contested [68,69].

Both cells were passed through a geometric verification before any energy was computed; it counts atoms, composition, the full bond-length distribution, the coordination histogram, the number of two-coordinate chain centers and the linearity of each chain, and fails on any contact below 1.35 Å. The verified cells were then relaxed, atomic positions and cell together, with the MACE-MP-0 foundation machine-learned interatomic potential [70], a message-passing equivariant network [71] trained on periodic density-functional data for inorganic solids, using the FrechetCellFilter and the Broyden–Fletcher–Goldfarb–Shanno (BFGS) optimizer as implemented in the Atomic Simulation Environment [55] to a maximum force of 0.02 eV Å^−1^. Relaxed lattice parameters are compared with experiment as the validation, and the geometric verification is repeated on the relaxed cells. That validation covers the lattice and the local geometry only; no elastic constants, phonons or formation energies were computed, and the potential’s description of a hydroxylated surface is outside the inorganic-crystal data it was trained on, which is why the surface B–O distance is anchored separately against a hybrid-functional calculation on $B(OH{)}_{3}$.

The surface used to test the molecular model is the (0001) termination of the relaxed $B_{4}C$ cell. Boron carbide has no plane perpendicular to **c** that passes through no icosahedron, so the cleavage height was chosen rather than assumed: the number of bonds cut was counted on a fine grid of candidate heights and the minimum taken, which cuts 4 bonds per cell against 12 for the worst plane. Fewest broken bonds is a proxy for stability and not a surface energy, which was not computed. Every atom left under-coordinated relative to its own coordination number in the bulk was then capped, boron with a hydroxyl and carbon with hydrogen, each cap placed in turn at the position furthest from every atom already present so that no cap lands on its neighbor. The slab is two bulk repeats thick with 12 Å of vacuum, with the lower third held fixed, and was relaxed with the same potential. The resulting hydroxyl coverage, 13 OH nm^−2^, is the ideal fully hydroxylated limit and is an upper bound, not an estimate of what a real powder carries. Miller indices in this system are setting-dependent, and (001) in the rhombohedral primitive setting is not the plane that (0001) denotes here; the $B_{4}C$ surface characterized most thoroughly from first principles is $\left(10\bar{1}1\right)$ [72], with a broader survey published recently [73].

A bond length is a test of local structure, not of energetics, so three further models test the interface chemistry directly. (i) *Amine–boron coordination.* A primary amine is a Lewis base and a three-coordinate boron a Lewis acid, and amines form dative N→B adducts with a wide range of boron Lewis acids [74]. The free energy of association of *n*-propylamine with boric acid and with boroxine, $B_{3}O_{3}(OH{)}_{3}$, was computed for the dative adduct and for the hydrogen-bonded alternative at 298 and 453 K (GFN2-xTB/ALPB with quasi-RRHO thermochemistry, 1 mol L^−1^ standard state), with PBE0-D3(BJ)/def2-SVP/IEF-PCM(*ε* = 3.4) single points as a check on the association energy. (ii) *Larger clusters.* Real boron carbide powder carries an oxidized, B–O-rich surface layer [75], whose structural unit in $B_{2}O_{3}$ is the boroxol ring. The monodentate graft was therefore rebuilt on a diborate, $H_{2}N(CH_{2}{)}_{3}Si(OH{)}_{2}-O-B(OH)-O-B(OH{)}_{2}$, which adds a B–O–B bridge and a neighboring hydroxylated site, and on a boroxol ring carrying two further B–OH sites, and both channels were taken through the full protocol of Section 2.4; the diborate pair was also re-optimized at the DFT level as described in Section 2.1, its substitution structure only to an approximate saddle point. (iii) *Water.* One and two water molecules were placed donating a hydrogen bond to the leaving chloride of the grafted-silane transition states, and both saddle points were re-optimized and tested against the acceptance criteria of Section 2.4.

### 2.10. Thermal-Neutron Shielding Estimate

Macroscopic cross sections were computed as $\Sigma =\sum_{i}N_{i}\sigma_{i}$ over the elements present, where $N_{i}$ is the number density of element *i* and $\sigma_{i}$ its microscopic absorption cross section, with microscopic cross sections at 2200 m s^−1^ from the compilation of Sears [76] and composite densities from the void-free inverse rule of mixtures. The absorption cross sections used are 0.3326 b (H), 0.0035 b (C), 33.5 b (Cl) and 767 b (natural B), with densities of 1.40 g cm^−3^ (PVC), 0.95 g cm^−3^ (HDPE), 0.92 g cm^−3^ (LDPE) and 2.52 g cm^−3^ ($B_{4}C$); compositions, number densities and the resulting cross sections are given in Table S3. Compilations differ on natural chlorine between about 33.5 and 35.5 b; the smaller value is used, which understates rather than overstates the effect claimed. The quantity computed is the macroscopic thermal-neutron absorption cross section of an infinite, homogeneous, void-free medium. No geometry, no neutron transport or moderation, no energy spectrum other than 2200 m s^−1^, no secondary gamma production, no self-shielding within filler particles and no porosity is included. It is a preliminary estimate of which matrix needs less absorber, not a prediction of shielding performance, which requires a Monte Carlo transport calculation of a real geometry and measurement.

### 2.11. What Was Varied

The quantities the result might depend on were varied deliberately, and the outcome of each is reported in Section 3.8. The electronic structure was varied in two ways. Every transition state of the eight amine/substrate pairs was located again, independently, with GFN1-xTB [77], which uses atomic point-charge electrostatics and a fixed-coefficient dispersion correction where GFN2 uses multipole electrostatics and self-consistent, density-dependent dispersion, so that the two are not variants of one another. In addition, PBE0-D3(BJ) single points were computed at every saddle point of these pairs and of the cluster models, and the saddle points of two pairs and of the diborate pair were re-optimized at that level (Section 2.1), the diborate substitution structure only to an approximate saddle point.

The environment and the thermochemistry were varied next. The dielectric was represented by twelve parameterized continua and by IEF-PCM at two permittivities (Section 2.6), and the vibrational entropy by the quasi-RRHO treatment and five hard floors (Section 2.5). Conformation was sampled with up to eleven starting structures per transition state (Section 2.4).

Finally, the model itself was varied. The concerted scan drives two coordinates on one fixed linear schedule between one fixed pair of endpoints, so the grafted-silane/dyad system was rerun with the leaving-group range shortened to 2.9 Å and lengthened to 3.6 Å, with 51 points instead of 26, and with the base lagging the leaving group. The dyad model was chosen because the ground-state energy increment per repeat unit converges by the tetramer (Section 3.3), but that is not the quantity reported, so the barrier itself was recomputed on the tetramer. The $T^{1}$, $T^{2}$ and $T^{3}$ grafted silanes were run at the saturated dyad, and larger clusters and explicit water tested the surface model (Section 2.9).

### 2.12. Reproducibility

Every table entry and figure value in this paper is read programmatically from the calculation output files; no number was transcribed by hand. Headline values quoted in the text are additionally re-read from the machine-readable output and matched against the manuscript by an audit script, so that a number changed in one place and not the other is caught rather than published. The same script checks that every reference in the list is cited and every citation is listed, that every equation, table and figure is cross-referenced, and that no raw Unicode superscript or subscript has been used where the document needs real formatting. Figure layout was checked automatically for overlapping text, and the palette for color-vision deficiency; in every figure the distinctions are additionally carried by marker shape, hatching, line style or a printed label.

## 3. Results

### 3.1. The Boron Carbide Substrate

**Figure 1:**
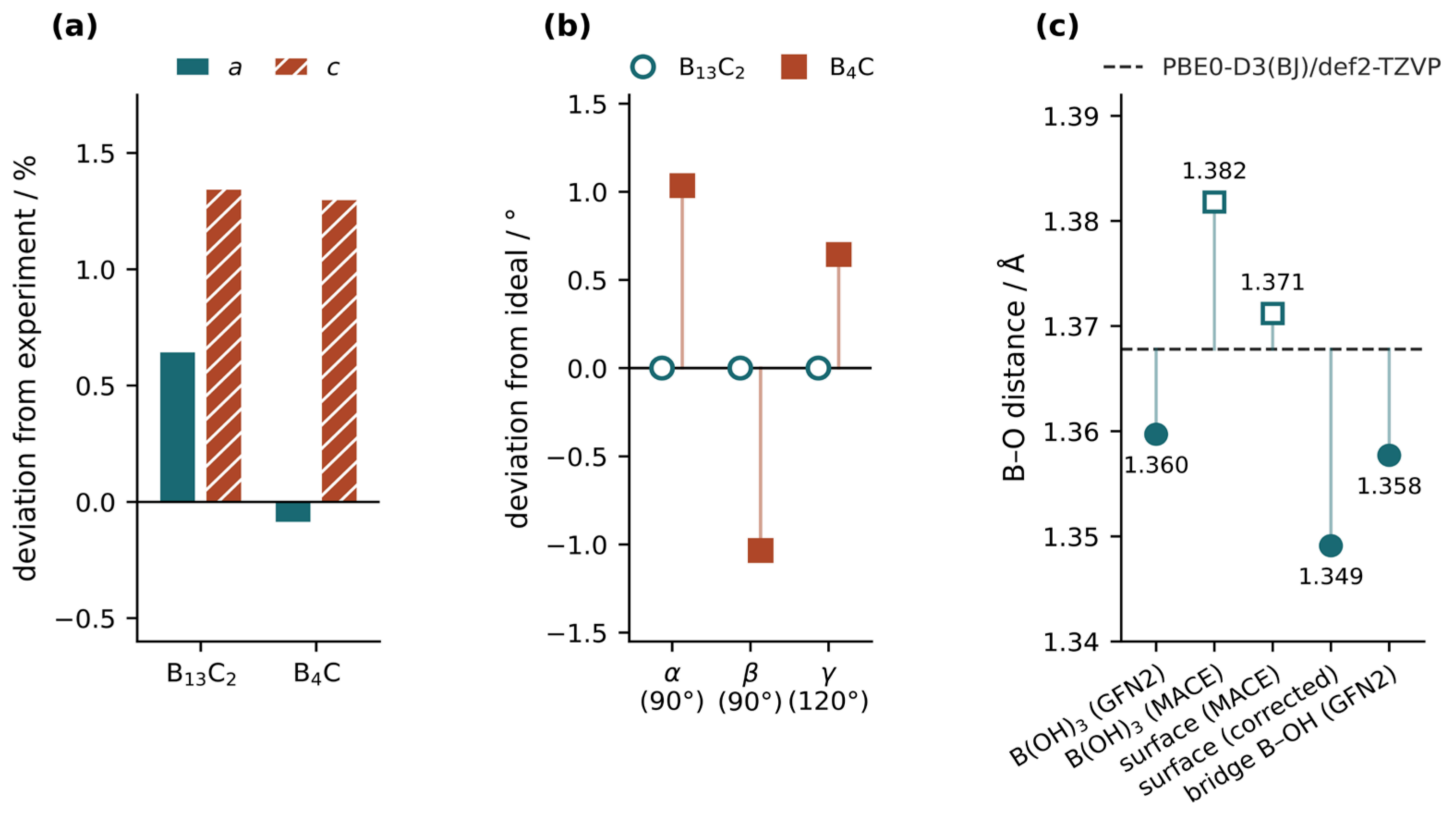
The boron carbide substrate and the interface model. (**a**) Deviation of the MACE-MP-0 relaxed lattice parameters from experiment (the refined cell of Kolel-Veetil et al. [65]) for the ordered $B_{13}C_{2}$ cell and for $B_{4}C$; the error is almost entirely in *c* and almost identical for the two compositions. (**b**) Relaxed cell angles: $B_{13}C_{2}$ stays hexagonal, while $B_{4}C$, whose polar icosahedral substitution is incompatible with $R\bar{3}m$, distorts spontaneously by about one degree. (**c**) The boron–oxygen distance in three environments, with PBE0-D3(BJ)/def2-TZVP on isolated $B(OH{)}_{3}$ as the reference (dashed line); the hydroxylated $B_{4}C(0001)$ surface value is shown as computed and after correction by the method offset measured on $B(OH{)}_{3}$, and lies within 0.01 Å of the cluster used for the interface chemistry.

The interface chemistry is computed on molecular models, so the solid filler is characterized first and the molecular model is then tested against it. Figure 1 summarizes what follows. Both ordered cells passed geometric verification before any energy was computed. The unrelaxed $B_{13}C_{2}$ cell contains 45 atoms as three formula units of $B_{12}(CBC)$: 36 six-coordinate icosahedral borons, six four-coordinate chain-end carbons and three two-coordinate chain-center borons, with intra- and inter-icosahedral B–B distances spanning 1.694 to 1.838 Å, an icosahedron-to-chain B–C distance of 1.590 Å, and three C–B–C chains of 1.433 Å at exactly 180.0°. The shortest contact anywhere in the cell is that chain bond.

Relaxation validates the model against experiment (Table 1). For $B_{4}C$ the relaxed *a* = 5.593 Å is 0.09% below the experimental 5.597 Å; *c* = 12.228 Å is 1.30% above 12.071 Å. For $B_{13}C_{2}$ the deviations are +0.64% and +1.34%. The error is thus almost entirely in *c* and almost identical for the two compositions, which identifies it as a systematic bias of the potential along the stacking direction rather than a defect of either structure. A 1.3% error in one axis is normal for a foundation potential used without fine-tuning and is small enough that the model supports the geometric statements made from it.

The symmetry lowering that Section 2.9 describes as a formal necessity is visible in the relaxed cell. $B_{13}C_{2}$, which is ordered in $R\bar{3}m$, relaxes with *α* = *β* = 90.0° and *γ* = 120.0° to within 10^−10^ degrees, i.e., it stays hexagonal. $B_{4}C$, with one polar icosahedral boron per icosahedron replaced by carbon, relaxes to *α* = 91.03°, *β* = 88.97° and *γ* = 120.64°. The cell does not merely fail to be $R\bar{3}m$ by construction; it spontaneously distorts away from hexagonal by about one degree when allowed to. This is a computed illustration of why an ordered $R\bar{3}m$ cell at $B_{4}C$ stoichiometry does not exist, and of why the site occupancies in experimental refinements are fractional.

The interface model can now be checked rather than assumed. The (0001) surface of the relaxed $B_{4}C$ cell was cleaved at the height that cuts the fewest bonds, four per cell against twelve for the worst choice, and hydroxylated. After relaxation the seven surface hydroxyls are strikingly uniform: B–O = 1.371 ± 0.002 Å across all seven, O–H = 0.982 Å, with B–O–H angles between 110° and 170°. The narrow spread in B–O against the wide spread in B–O–H is itself informative: the bond length is set by the local boron–oxygen chemistry and the angle by whatever hydrogen bonding the neighboring hydroxyls happen to permit, which is precisely the separation a cluster model relies on.

Table 2 puts the three descriptions of a boron–oxygen bond on one axis, with PBE0-D3(BJ)/def2-TZVP on $B(OH{)}_{3}$ as the anchor so that neither cheap method is used to judge the other. Against that anchor GFN2-xTB is 0.008 Å short and the foundation potential 0.014 Å long, both small and, more usefully, both systematic. Correcting the surface value by the 0.022 Å offset between the two cheap methods puts the hydroxylated surface at 1.349 Å on a GFN2 footing, against 1.358 Å for the B–OH of the grafted bridge and 1.360 Å for boric acid: agreement to 0.009 and 0.011 Å respectively, which is smaller than the 0.02 Å spread among the three inequivalent B–O bonds within $B(OH{)}_{3}$ itself.

With respect to the precision these methods resolve, the boron–oxygen bond of the cluster is therefore the bond a hydroxylated boron carbide surface presents to an approaching silanol. This is a test of local structure, not of energetics: the cluster does not contain neighboring surface sites, surface constraint or the underlying icosahedral network. Multidentate attachment, larger boron–oxygen clusters, explicit water and amine–boron coordination, which test the energetics directly, are reported in Section 3.7.

### 3.2. What the Composite Is Worth as a Shield

**Figure 2:**
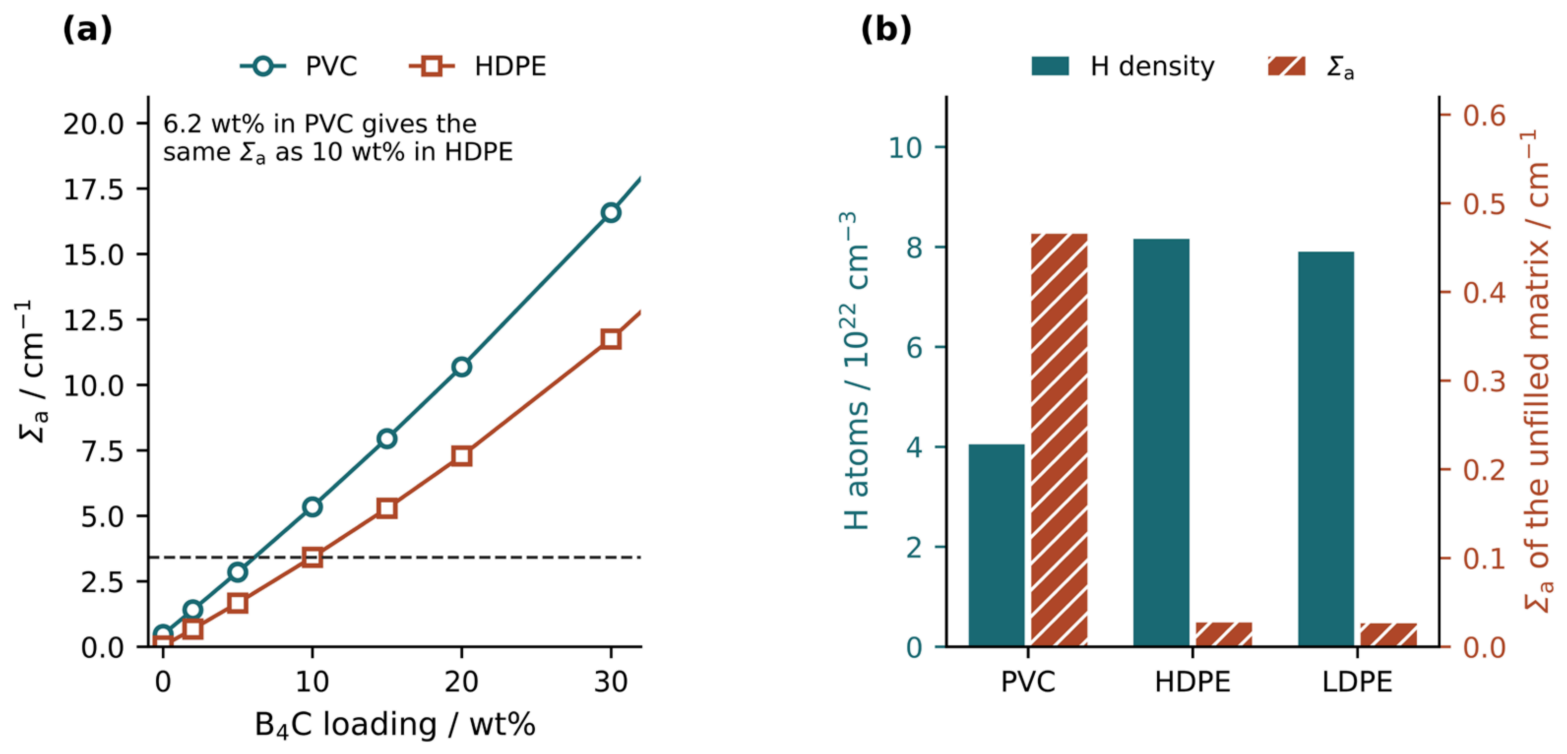
What the composite is worth as a shield. (**a**) Macroscopic thermal-neutron absorption cross section against $B_{4}C$ loading for the two candidate matrices; the dashed line marks the capture of high-density polyethylene at 10 wt%, which PVC reaches at 6.2 wt%. (**b**) The two competing properties of the unfilled matrices: hydrogen atoms per unit volume, which sets the moderating power (left axis, solid bars), and the absorption cross section of the matrix itself (right axis, hatched bars). PVC holds half the hydrogen of polyethylene and captures seventeen times as strongly. Microscopic cross sections at 2200 m s^−1^ are from the compilation of Sears [76]; densities and the mixing rule are given in Section 2.10 and Table S3. This is a preliminary, transport-free estimate.

The shielding rationale of the Introduction is quantified here, within the preliminary cross-section estimate defined in Section 2.10, because it determines how much filler, and therefore how much coupling agent, the composite needs (Figure 2). PVC holds 4.05 × 10^22^ hydrogen atoms per cm^3^ against 8.16 × 10^22^ for high-density polyethylene: 49.6%, less than half. Per unit volume, PVC is therefore a weaker moderator than the polyolefins the boron-composite literature uses. Against that, the chlorine is a genuine thermal absorber. An unfilled PVC has $\Sigma_{a}$ = 0.466 cm^−1^ against 0.027 cm^−1^ for unfilled HDPE, seventeen times as much, and that chlorine is worth 1.37 wt% of $B_{4}C$ added to HDPE.

The consequence for absorption favors PVC. Under the assumptions of Section 2.10, the macroscopic thermal-neutron absorption cross section of HDPE carrying 10 wt% $B_{4}C$ is reached with 6.2 wt% $B_{4}C$ in PVC, 3.8 percentage points less filler. Less filler means less interfacial area, less coupling agent, and, if the argument of the following sections holds, less of the interfacial degradation an aminosilane may cause. The trade is real but not free: the moderating power has to come from elsewhere in the shield design, and the chlorine that supplies the capture also emits secondary capture gammas that a practical design must account for. The estimate also omits neutron transport, the energy spectrum of a real source, self-shielding within filler particles and porosity; it identifies which matrix needs less absorber and is not a prediction of shielding performance.

### 3.3. Molecular Models

Oligomer length converges quickly. The energy increment per added repeat unit, $\left[E\left(n=4\right)-E\left(n=2\right)\right]/2$ versus $\left[E\left(n=6\right)-E\left(n=4\right)\right]/2$, changes by 0.568 kJ mol^−1^ between the two intervals, so an oligomer beyond the tetramer adds essentially nothing to the local electronic description of a C–Cl unit. That is a statement about ground-state energies and not about barriers, which represent a different quantity; Section 3.8 tests the barrier itself against chain length. Stereosequence dependence is small but not negligible: across the three atactic hexamers the relative electronic energies span 8.07 kJ mol^−1^, with *RRSSRS* lowest, *RSSRSR* 3.53 kJ mol^−1^ higher and *RSRSRS* 8.07 kJ mol^−1^ highest. Table 3 collects the species and their computed properties.

One entry in Table 3 anticipates the rest of the paper. The saturated PVC models have gaps between the highest occupied and lowest unoccupied molecular orbitals (HOMO–LUMO) of 6.23–6.73 eV, decreasing slightly with chain length. The allylic chloride has a gap of 4.86 eV, a drop of 1.87 eV caused by the π system introduced by the newly formed double bond. That collapse is the electronic signature of the first step of polyene formation, and it is consistent with both the reactivity change of Section 3.9 and the bond weakening of Section 3.10.

### 3.4. Grafting Is Thermodynamically Accessible

**Figure 3:**
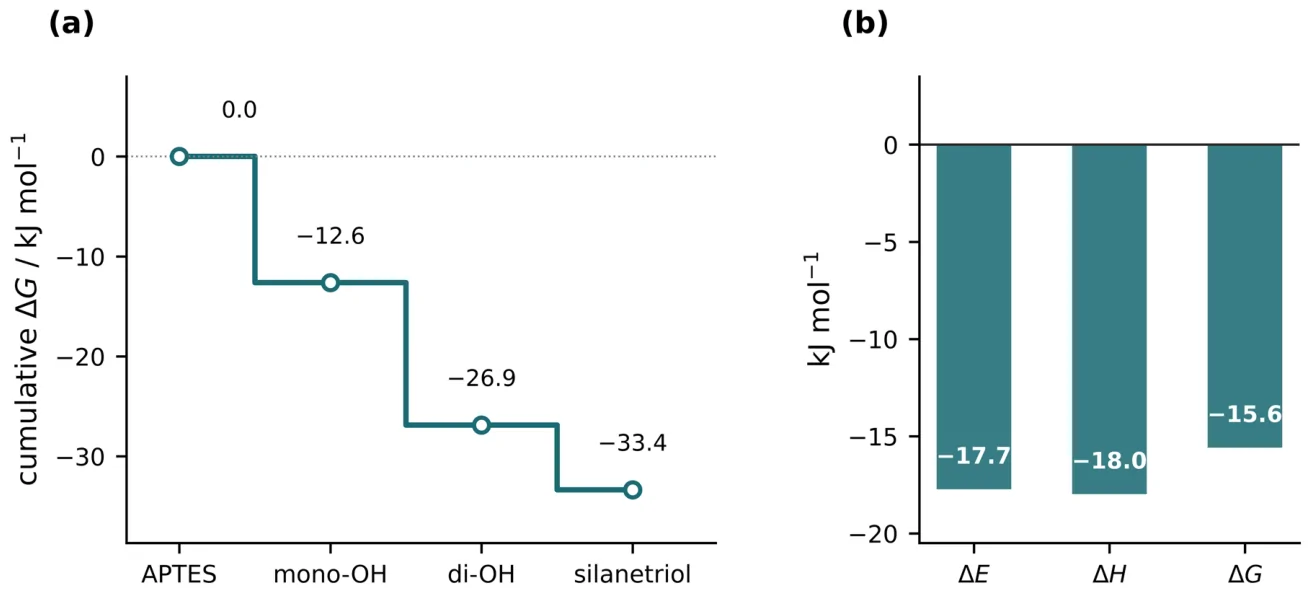
Grafting thermodynamics at 298.15 K. (**a**) Cumulative free energy along the stepwise hydrolysis of APTES to 3-aminopropylsilanetriol, Equation (5); abscissa labels identify the silane after each step and annotations give the cumulative ΔG in kJ mol^−1^. (**b**) Electronic energy, enthalpy and free energy of the condensation of Equation (6), in which boric acid represents a hydroxylated surface boron site.

The reactive form of APTES is not the parent triethoxysilane but its hydrolysis products. The three sequential steps of Equation (5), with *n* = 0, 1 and 2,

(5) $$R-Si{\left(OEt\right)}_{3-n}{\left(OH\right)}_{n}+H_{2}O\;\to \;R-Si{\left(OEt\right)}_{2-n}{\left(OH\right)}_{n+1}+EtOH,\;R={\left({CH}_{2}\right)}_{3}{NH}_{2}$$

are each exergonic at 298.15 K: −12.63, −14.24 and −6.48 kJ mol^−1^, giving −33.35 kJ mol^−1^ overall for complete conversion to 3-aminopropylsilanetriol (Figure 3a). The magnitudes are modest, as expected when one Si–O bond is exchanged for another. Under any humid processing condition the silanetriol is therefore accessible, and modeling only intact APTES would treat the least reactive member of the series as though it were the relevant one.

Grafting proceeds by condensation of a surface B–OH with a silanol, as in Equation (6):

(6) $$B{\left(OH\right)}_{3}\;+\;{\left(HO\right)}_{3}Si-{\left({CH}_{2}\right)}_{3}{NH}_{2}\;\to \;{\left(HO\right)}_{2}B-O-Si{\left(OH\right)}_{2}-{\left({CH}_{2}\right)}_{3}{NH}_{2}\;+\;H_{2}O$$

This is favorable by ΔE = −17.73, ΔH = −17.98 and ΔG = −15.59 kJ mol^−1^ (Figure 3b); zero-point and thermal corrections reduce the exergonicity by about 2 kJ mol^−1^, the entropy penalty of forming one molecule from two being partly offset by the released water. Two limitations attach. The model captures the local bond exchange but not surface constraint, site heterogeneity or cooperativity between neighboring grafted silanes, though multidentate attachment is treated explicitly in Section 3.7. And because water is released, the position of the equilibrium depends on the water activity of the medium. What the result establishes is sufficient for what follows: Si–O–B formation is not forbidden, so the grafted state whose reactivity is examined next is a real state.

### 3.5. From Scan Maxima to Transition States

**Figure 4:**
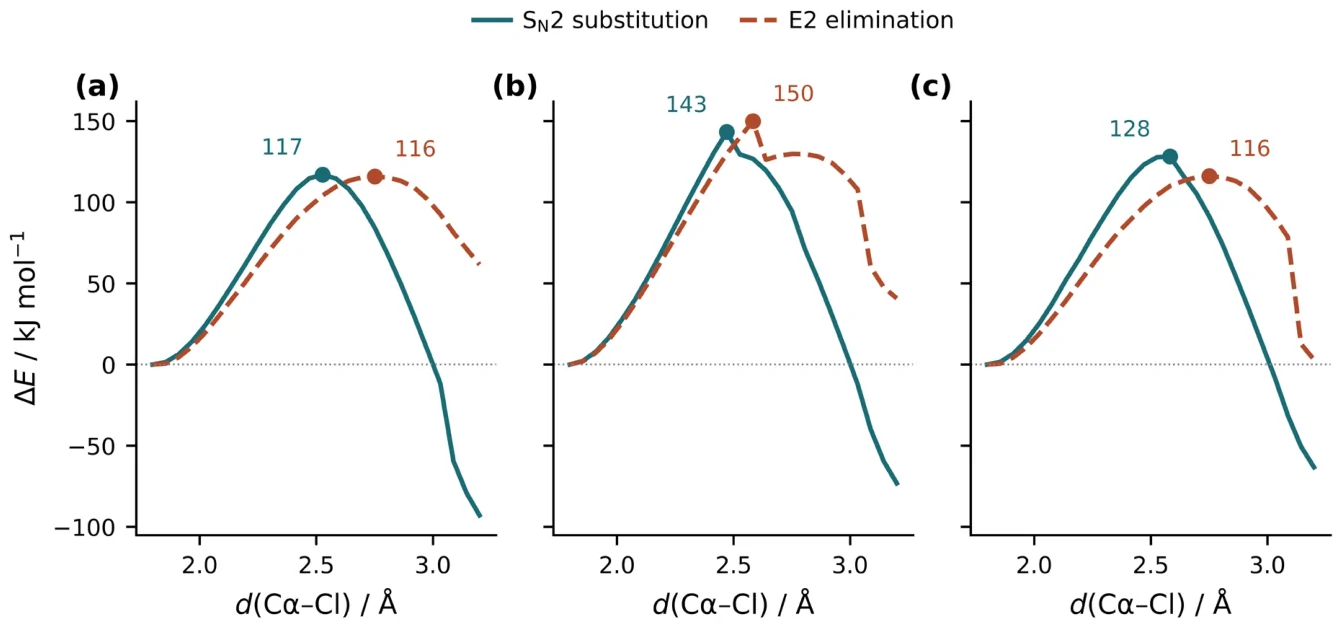
The relaxed scans from which the transition-state searches were started, at the saturated PVC dyad. Energies for nucleophilic substitution (teal, solid) and E2 elimination (rust, dashed) are plotted against the shared leaving-group coordinate *d*(Cα–Cl); filled circles mark the profile maxima and the adjacent numbers give the constrained maximum in kJ mol^−1^. (**a**) *n*-Propylamine, a minimal aminopropyl model without the silanol head group. (**b**) 3-Aminopropylsilanetriol, the fully hydrolyzed pre-grafting species. (**c**) The monodentate B–O–Si bridged species representing the grafted state. GFN2-xTB with ALPB implicit solvation (chloroform, *ε* = 4.7). These are constrained maxima on a two-coordinate scan, not saddle points; Section 3.5 shows how far each sits from the transition state it stands in for, and no branching is inferred from this figure.

The competing paths were first mapped by relaxed scans, and Figure 4 shows those profiles. They are useful as a picture of the two mechanisms and as a source of starting geometries, and they are not used for anything else, because a maximum along a two-coordinate concerted scan is a constrained maximum and need not be a saddle point at all. Each was therefore refined on the unconstrained surface and tested.

Twenty-two searches were attempted under GFN2-xTB, not counting the four searches on the cluster models of Section 3.7 that Table S2 also lists. Nineteen converged to structures that pass every condition of Section 2.4. Two more, the pair used for the chain-length test of Section 3.8, converged to structures carrying a second imaginary mode of −11.5 and −44.9 cm^−1^ besides the reaction mode; both are hindered silanol rotors rather than second reaction coordinates, and those two structures are used with that flag, their soft mode being treated in the quasi-RRHO scheme of Section 2.5. One search, the substitution channel of the base-lagging scan schedule, returned a third-order saddle and is reported as a failure rather than repaired. Under GFN1-xTB, fourteen of sixteen searches were accepted, and the remaining two, the elimination channels of *n*-propylamine and of the monodentate graft at the saturated dyad, carry a second mode of −27 and −22 cm^−1^ of the same hindered-rotor kind and are used with the same flag. Conformational sampling (Section 2.4) then started 160 further searches from the 16 GFN2-xTB transition states of Table 4; 124 of them passed the acceptance test, and together with the original structures they give 136 distinct transition-state conformers, listed with their outcome in Table S2.

Under GFN2-xTB the imaginary frequencies separate the two channels cleanly: elimination transition states have modes between −962 and −1262 cm^−1^, the signature of a hydrogen in flight between nitrogen and carbon, while substitution transition states have modes between −410 and −539 cm^−1^, the heavier motion of a chloride leaving as a nitrogen closes in. There is no overlap between the two distributions and no ambiguity about which channel a given saddle belongs to. Overlaps between the imaginary mode and the intended reaction coordinate run from 0.34 to 0.70. RMSD values from the scan maxima range from 0.08 to 1.53 Å, which is a direct measure of how far a constrained scan maximum can sit from the saddle it is standing in for.

The difference between the two comparisons is large, and it runs in one direction. Table 4 lists, for each amine–substrate pair, the barrier difference obtained from the scan maxima, with each barrier measured from its own encounter complex, and then the same quantity taken between the two transition-state energies, which requires no reference state because the two structures have identical composition. Two separate effects contribute.

The first is the reference state. Measuring each barrier from its own encounter complex compares two numbers with two different zeros, and the two complexes differ in energy by up to 10.5 kJ mol^−1^ because they were assembled differently. Since the complexes interconvert far faster than either reacts, the Curtin–Hammett principle makes that comparison the wrong one; the right one is the difference of transition-state energies. Together with the relaxation from each scan maximum to the true saddle point, removing the mismatch changes $\Delta \Delta E^{‡}$ by 1.3 to 37.0 kJ mol^−1^ across the eight pairs of Table 4, always toward substitution, and reverses its sign for *n*-propylamine and for the tridentate graft at the saturated dyad.

The second is larger, and a comparison of electronic energies cannot show it, because one channel transfers a proton and the other does not. In the elimination transition state a C–H stretching mode of order 2900 cm^−1^ has become the reaction coordinate and left the zero-point sum; in the substitution transition state the mode that leaves is a C–Cl stretch six times lower in frequency. The elimination transition state therefore loses far more zero-point energy, and the difference is not small: across the ten pairs computed with GFN2-xTB (the eight of Table 4 and the dyad and tetramer pairs of the chain-length test in Section 3.8) the zero-point and thermal term shifts the barrier difference by +12.4 kJ mol^−1^ on average, between +11.5 and +13.4, always toward elimination. Entropy adds a further +6.3 kJ mol^−1^ on average and up to +9.8, because the elimination transition state is the looser of the two, with the C–Cl bond extended further. Translational contributions cancel exactly for two structures of identical composition, and rotational contributions differ by at most 1.5 kJ mol^−1^; both are computed rather than assumed. Tunneling then adds a final asymmetry, with Wigner factors of 1.39–1.66 for elimination against 1.07–1.12 for substitution.

**Figure 5:**
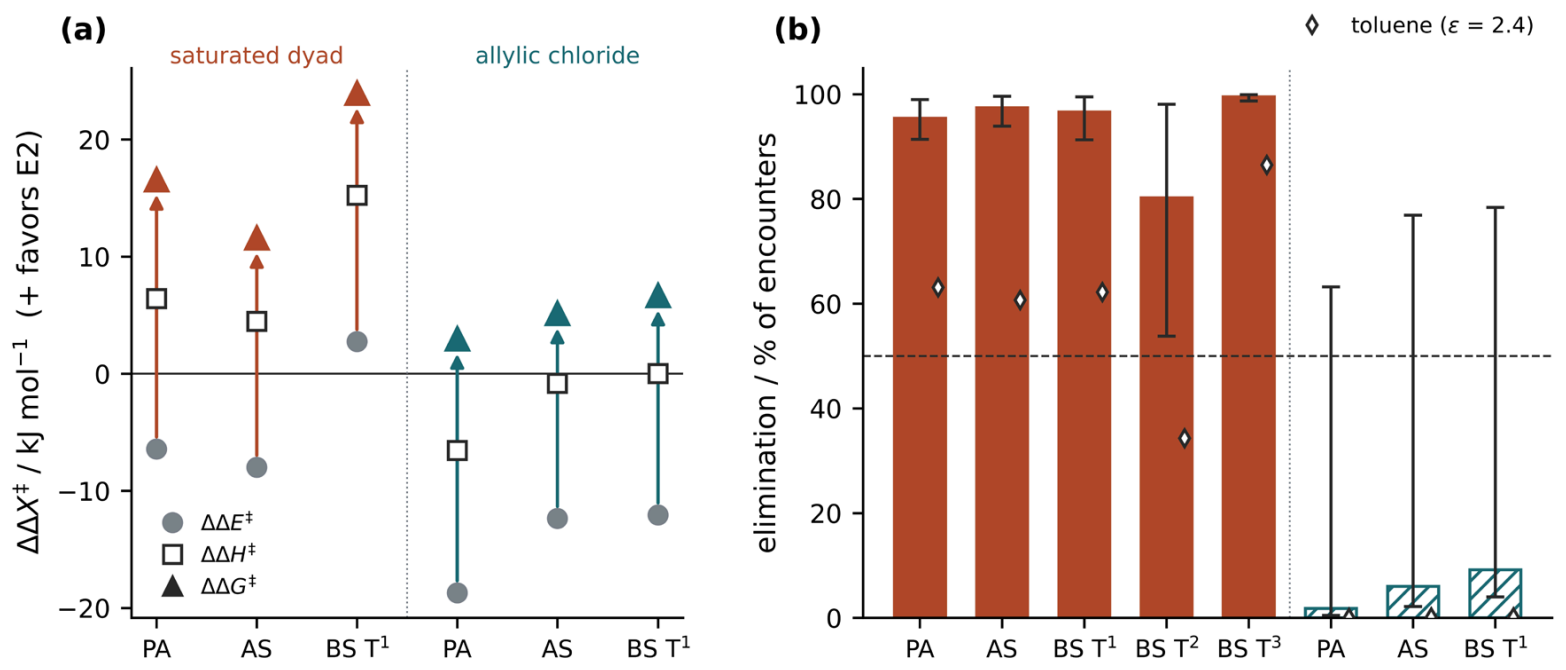
Where the semiempirical selectivity comes from, and what it amounts to. (**a**) The three barrier differences of Equation (1) under GFN2-xTB in chloroform for the three amine models at the saturated PVC dyad and the same three at the allylic chloride; positive favors elimination. Gray circles are $\Delta \Delta E^{‡}$, open squares add zero-point and thermal corrections to give $\Delta \Delta H^{‡}$, and filled triangles add entropy to give $\Delta \Delta G^{‡}$ (quasi-RRHO, 453 K). The arrow on each system runs from the electronic to the free-energy value: the correction that overturns the electronic preference exists because only the elimination transition state carries a hydrogen in flight. (**b**) Fraction of reactive encounters entering the elimination channel at 453 K, from the conformer-summed form of Equation (2) with Wigner tunneling. Bars are the median over the six ALPB solvents of the dielectric band (2.9 ≤ *ε* ≤ 8.9), whiskers give the range, and diamonds mark the value in toluene (*ε* = 2.4). Solid rust bars are the saturated dyad, hatched teal bars the allylic chloride, and the dashed line marks equal partitioning. The upper whiskers of the allylic bars are the chloroform values, which lie off the trend of the other solvents (Figure 6a). The density functional result, which reverses the majority channel at the saturated dyad, is given in Table 5.

At the GFN2-xTB level the sum of these is decisive. Across the ten pairs the electronic barrier difference favors substitution in eight, by as much as 18.7 kJ mol^−1^, and the free-energy difference favors elimination in all ten of them in chloroform (Figure 5a shows six of them); Section 3.6 gives the fraction over the dielectric band. The channel that wins is not the one with the lower electronic barrier. It wins on zero-point energy and entropy, and it wins because it is the channel with a hydrogen in flight.

### 3.6. Selectivity at a Saturated Backbone Unit: Semiempirical Result and DFT Validation

Figure 5b converts the conformer-summed free-energy differences into the fraction of reactive encounters that eliminate, through Equation (2), over the dielectric band of Section 2.6. Under GFN2-xTB, at a saturated backbone C–Cl unit, elimination is the majority channel for every amine model and every attachment mode throughout the band. The band median is 95% for the bare aminopropyl model, 97% for the free silanetriol and for the monodentate graft, 80% for the bidentate graft and more than 99% for the tridentate graft, and the lowest value anywhere in the band is 54%, for the bidentate graft. Under GFN1-xTB the same systems give band medians of 96 to 100% and a lowest value of 70%. The two Hamiltonians differ in the ordering within this set but agree on the majority channel: whatever state the aminosilane is in, when its amine meets a saturated C–Cl unit of PVC, the dominant outcome at the semiempirical level is loss of HCl, not formation of a C–N bond. The last part of this section tests that result against density functional theory.

The band does not cover every environment. In the two ALPB solvents below it, toluene and carbon disulfide (*ε* = 2.4 and 2.6), elimination falls to 43–63% for *n*-propylamine, the free silanetriol and the monodentate graft, to 12–34% for the bidentate graft and to 84–87% for the tridentate graft (toluene values in Figure 5b). In a nonpolar environment the two channels are therefore close to parity, and the semiempirical preference for elimination holds for the melt and a polar interphase rather than for a nonpolar one. The grafting state itself has a smaller effect: the band medians of the free silanetriol and of the monodentate graft differ by less than one percentage point, and the apparent inversion of selectivity on grafting that barriers measured from their own encounter complexes would suggest disappears when the transition states are compared directly.

Whether the reaction is fast enough to matter during processing follows from the absolute barriers, measured from the separated reactants and converted to 1 mol L^−1^ (Section 2.5). For the elimination channel of the monodentate graft the conformer-summed activation free energy is 139 to 161 kJ mol^−1^ over the band (median 154), and for the bare aminopropyl model 128 to 148 kJ mol^−1^ (median 144). With the C–Cl concentration of PVC, the half-life of a grafted amine at 453 K, with both channels included, lies between 33 s and 3.2 h, with a band median of 32 min, and that of the bare amine between 2 s and 5 min. At the semiempirical level these times are comparable to melt-processing times (Section 2.5); the absolute values carry the larger uncertainty of absolute semiempirical barriers, and the density functional values are given below.

Density functional theory does not confirm the semiempirical preference. For *n*-propylamine and for the monodentate graft at the saturated dyad, both transition states were re-optimized at PBE0-D3(BJ)/def2-SVP/IEF-PCM(*ε* = 3.4) and verified on that surface (Section 2.1, Table 5). The substitution saddle points are nearly unchanged from GFN2-xTB: they lie 0.05 and 0.25 Å (RMSD) from the GFN2-xTB structures, with imaginary frequencies of −426 and −444 cm^−1^, and for *n*-propylamine the reaction mode overlaps the GFN2-xTB one to 0.997. The elimination saddle points change character. Under GFN2-xTB the proton is in flight between carbon and nitrogen (N···H 1.30 Å, C···H 1.38 to 1.39 Å); at the DFT level it has already moved to the nitrogen (N–H 1.13 to 1.14 Å, C···H 1.65 to 1.72 Å) while the C–Cl bond is still 2.33 to 2.36 Å long, so that the remaining motion is chloride departure, with a curvature of only −125 to −128 cm^−1^. The elimination is therefore strongly asynchronous, an E2 transition state with far-advanced proton transfer, approaching the limit of elimination through the conjugate base (E1cB), rather than a synchronous one. Because these elimination modes are soft and the curvature was checked only in a subspace, the four saddle points were also connected to their two sides by downhill relaxations at the same level, as described in Section 2.1, with the end points listed in Table S11. From each substitution saddle point, one direction restores the C–Cl bond (1.87 Å) with the nitrogen withdrawn to 2.77 to 2.96 Å, 63 to 78 kJ mol^−1^ below the saddle point in electronic energy, and the other forms the C–N bond (1.56 to 1.59 Å) and expels the chloride to 3.5 to 4.1 Å, 87 to 89 kJ mol^−1^ below it, at the points where the relaxations were stopped. From each elimination saddle point, one direction returns the proton to the β-carbon (C–H 1.10 to 1.11 Å) and restores the C–Cl bond (1.81 to 1.82 Å), 130 to 131 kJ mol^−1^ below the saddle point, and the other lengthens the C···Cl distance to 2.85 to 2.88 Å, past the 2.8 Å threshold that Table S11 uses to assign the product side, with the alkene formed (C=C 1.35 Å) and the proton on the nitrogen. On this side the energy had fallen by only 7 to 8 kJ mol^−1^ where the relaxations were stopped, which is consistent with the soft reaction mode and with a late, product-like saddle point. The soft imaginary modes therefore belong to the intended elimination. These relaxations establish on which side of each saddle point the reactants and the products lie; they do not trace the minimum-energy path and are not intrinsic reaction coordinate calculations.

This change of character removes most of the advantage that decides the semiempirical result. With the reacting core described at the DFT level, the zero-point difference in favor of elimination for *n*-propylamine falls from 16.4 kJ mol^−1^ under GFN2-xTB to 7.2 kJ mol^−1^, and for the grafted silane from 16.0 to 7.0 kJ mol^−1^, because the transferred proton now vibrates as a real N–H mode rather than forming the reaction coordinate, and the Wigner factor of the elimination channel falls to near unity. The electronic barrier difference, meanwhile, favors substitution by 23.7 to 28.8 kJ mol^−1^ for *n*-propylamine across two basis sets and three functionals, and by 19.1 to 30.5 kJ mol^−1^ for the grafted silane. The resulting free-energy difference is −15 to −19 kJ mol^−1^ for *n*-propylamine and −12 to −19 kJ mol^−1^ for the grafted silane, the range spanning the two basis sets, so that elimination accounts for roughly 1% of reactive encounters at 453 K and at most a few percent (at face value, 0.6 to 1.7% and 0.6 to 3.9%, respectively). These fractions rest on one transition-state conformer per channel and on the thermal terms from the mixed DFT/GFN2-xTB Hessians of Section 2.1; as Section 4.5 sets out, they are order-of-magnitude estimates, not a product ratio for a real composite.

The semiempirical preference for elimination is therefore not reproduced by the hybrid functional, and it most likely arises from the way the GFN Hamiltonians describe the proton transfer rather than from the chemistry. Both GFN2-xTB and GFN1-xTB (under GFN1-xTB, N···H 1.29 to 1.31 Å and C···H 1.33 to 1.35 Å at the saturated dyad) describe the elimination as a synchronous proton transfer, and the zero-point advantage that follows from that description is large enough to overturn an electronic preference for substitution; the hybrid functional places the proton transfer before the transition state and leaves the electronic preference in place. At the level of theory on which the conclusions of this work rest, and for these two model pairs, the pendant amine reacts with a saturated C–Cl unit predominantly by substitution, which forms a C–N bond to the polymer and converts the amine to its hydrochloride. Elimination remains a minor channel, but every elimination event leaves an allylic chloride, whose consequences are examined in Section 3.9 and Section 3.10.

The density functional barriers measured from the separated reactants (Table 5) set how fast an amine is consumed. For *n*-propylamine the substitution barrier is 147 to 156 kJ mol^−1^ (1 mol L^−1^, 453 K; def2-SVP and def2-TZVP energies) and the elimination barrier 166 to 171 kJ mol^−1^, so that the amine has a half-life of 5 to 43 min in the melt. For the grafted silane the substitution barrier is 159 to 175 kJ mol^−1^, close to the 159 to 171 kJ mol^−1^ of the conformer-summed GFN2-xTB value over the band, and the elimination barrier 179 to 186 kJ mol^−1^; the half-life of a grafted amine is 2 h to 4 days. At the semiempirical level the grafted amine was consumed within about half an hour mainly because its elimination barrier was low, and that is the channel density functional theory removes. These are single transition-state conformers; at the GFN2-xTB level, summing over the conformer ensembles lowered the absolute barriers of these two pairs by 3 to 8 kJ mol^−1^, which would shorten the half-lives by up to an order of magnitude. A free amine is therefore consumed on the time scale of melt processing, whereas the fraction of grafted amines that reacts within 20 min at 453 K ranges from about 0.2% to about two thirds across these estimates, and the calculations do not decide whether grafted amines react to a significant extent during processing. All of these half-lives are order-of-magnitude estimates. They treat the reaction as a bimolecular encounter in a homogeneous melt at the C–Cl concentration of bulk PVC, with a standard state of 1 mol L^−1^, and they assume that a grafted amine reaches C–Cl units as freely as a free one; at a real interface the tether, the local chain density and the fraction of amines within reach of the polymer will change them, in a direction the calculations do not fix.

### 3.7. The Attachment Mode and Larger Interface Models

The attachment mode was varied because the free silanols of a graft can hydrogen-bond to the leaving chloride, and because aminosilanes on oxide surfaces sit mostly as $T^{2}$ and $T^{3}$ rather than $T^{1}$ [16]. The answer is that it matters little. Over the dielectric band the GFN2-xTB ensemble medians are 97% ($T^{1}$), 80% ($T^{2}$) and more than 99% ($T^{3}$), and the GFN1-xTB medians 100%, 98% and 97%. No attachment mode brings elimination below the majority within the band under either Hamiltonian; the lowest band values are 54% and 70%, both for the bidentate graft. The reading consistent with both Hamiltonians is that the number of surviving silanols modulates the effect at the margin without changing its direction, and that the attachment mode is not a lever on the selectivity under either semiempirical Hamiltonian.

Three further models test whether the single boron site misrepresents the interface chemistry (Section 2.9, Table S10). On the diborate, which adds a B–O–B bridge and a neighboring B–OH site, both channels of the monodentate graft gave verified transition states (the substitution one with a soft second mode of −15 cm^−1^ of the hindered-rotor kind), and $\Delta \Delta G^{‡}$ is 44 to 64 kJ mol^−1^ over the band, so that elimination accounts for essentially all encounters. On the boroxol ring the elimination saddle point is well defined, but the substitution saddle point has an imaginary frequency of only −180 cm^−1^ and an overlap of 0.26 with the reaction coordinate, both close to the acceptance limits of Section 2.4; its much larger $\Delta \Delta G^{‡}$ of 63 to 89 kJ mol^−1^ is therefore reported but not relied on. At the GFN2-xTB level, neither larger cluster moves the selectivity toward substitution. One water molecule donating a hydrogen bond to the leaving chloride leaves the selectivity of the monodentate graft unchanged, with 98 to 100% elimination over the band (both transition states carry a soft second mode, of −20 and −17 cm^−1^, of the hindered-rotor kind); with two water molecules the searches ended on structures carrying additional imaginary modes of up to 75 cm^−1^, and no value is reported for them.

PBE0-D3(BJ)/def2-SVP single points at the GFN2-xTB saddle points move the electronic barrier difference of the monodentate graft from −54.4, −29.9 and −20.9 kJ mol^−1^ on the single boron site (gas phase and IEF-PCM at *ε* = 3.4 and 7.4) to −3.3, +5.4 and +7.6 kJ mol^−1^ on the diborate, a shift of 28 to 51 kJ mol^−1^ toward elimination; on the boroxol ring the same single points give +47.5 kJ mol^−1^ at *ε* = 3.4, although the substitution saddle point of that model is marginal. These single points do not survive re-optimization. When both transition states of the diborate pair are re-optimized at PBE0-D3(BJ)/def2-SVP/IEF-PCM(*ε* = 3.4), the hydrogen bond from a boron hydroxyl of the cluster to the leaving chloride in the substitution transition state, already present but longer in the GFN2-xTB geometry, tightens (H···Cl shortened from 2.46 to 2.19 Å), and the electronic barrier difference becomes −40 kJ mol^−1^, again favoring substitution (single boron site: −26.4 kJ mol^−1^). The elimination saddle point, taken at a residual force of 0.046 eV Å^−1^, is verified on the continuum surface, with one negative curvature (−110 cm^−1^) and the same asynchronous character as on the single site. The substitution search did not converge, and its structure is treated here as an approximate saddle point rather than a converged transition state: its energy stopped changing at a residual force of about 0.46 eV Å^−1^, 2.6 kJ mol^−1^ below the structure used here, which is the point of lowest residual force along the search (0.17 eV Å^−1^) and which has, in the subspace test of Section 2.1, one negative curvature (−405 cm^−1^, overlap 0.97 with the GFN2-xTB reaction mode). Taking the lower-energy end point instead would make the barrier difference about 3 kJ mol^−1^ more negative. With the mixed Hessian of Section 2.1, the thermal term of the diborate pair is +8.8 kJ mol^−1^ (zero-point term +5.1 kJ mol^−1^), close to the +7.2 and +8.7 kJ mol^−1^ of the single-site and propylamine pairs, and $\Delta \Delta G^{‡}$ at 453 K is −31 kJ mol^−1^ with def2-SVP; because the substitution structure is not converged, this value indicates the sign of the preference and not its size. No def2-TZVP energy was computed for this pair; the basis-set change that raised the barrier difference of the single-site and propylamine pairs by 4 to 7 kJ mol^−1^ would not change its sign. The mixed Hessian of the substitution transition state carries a second negative mode of −97 cm^−1^, larger in magnitude than any extra mode of the single-site substitution transition state (at most 48 cm^−1^), which adds to the uncertainty of the thermal term. Replacing the extra negative modes of both transition states by 50, 100 or 150 cm^−1^, or omitting them, as in the sensitivity test of Section 4.5, changes the thermal term of this pair by at most 3.8 kJ mol^−1^, which does not change the sign. Within these limits, the diborate gives no indication that the density functional preference is reversed, but the size of the effect shows that single points at semiempirical geometries are not reliable on these clusters, and that the result depends on the hydrogen-bond pattern between the cluster and the leaving chloride, which was sampled only at the GFN2-xTB level.

Coordination of the amine to boron was tested because it would take amines out of reach of the polymer. With boric acid no dative adduct forms: the N–B distance relaxes to 3.5 Å and the complex is held by hydrogen bonds only; the separately built hydrogen-bonded complex retains one soft mode of −24 cm^−1^, treated as a low-frequency vibration. With boroxine, $B_{3}O_{3}(OH{)}_{3}$, a dative N→B adduct forms with an N–B distance of 1.75 Å. At the GFN2-xTB level, neither this adduct nor any hydrogen-bonded complex is bound in free energy at a standard state of 1 mol L^−1^ (ΔG = +4 to +22 kJ mol^−1^ at 298 K and +26 to +46 kJ mol^−1^ at 453 K). PBE0-D3(BJ) binds all complexes more strongly, by 23 to 54 kJ mol^−1^ in the electronic association energy; with the GFN2-xTB thermal terms this makes the boroxine adduct bound by about 32 kJ mol^−1^ at 298 K and by about 8 kJ mol^−1^ at 453 K, an estimate that the small basis, without counterpoise correction, biases toward binding. Amines may therefore be partly coordinated to three-coordinate boron at an oxide-rich surface after cooling, but at the processing temperature the coordination is weak and reversible, and it does not prevent an uncoordinated amine from reacting with the polymer.

### 3.8. Robustness

**Figure 6:**
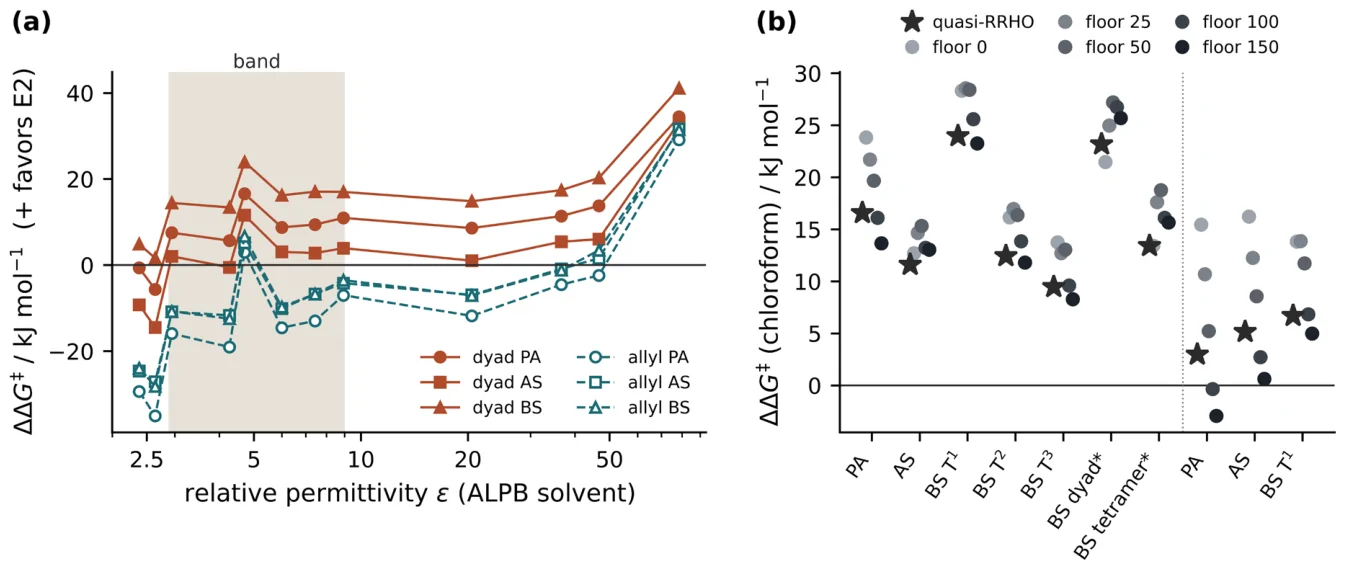
Robustness of the semiempirical barrier difference. (**a**) $\Delta \Delta G^{‡}$ of the original transition-state pair against the relative permittivity of the ALPB continuum (single points at the chloroform geometries, quasi-RRHO thermal term) for the three amine models at the saturated dyad (solid, rust) and at the allylic chloride (dashed, teal); the shaded region is the band of Section 2.6. Each ALPB solvent carries its own parameters, so the lines only guide the eye and do not represent a function of *ε*. (**b**) $\Delta \Delta G^{‡}$ in chloroform under the quasi-RRHO treatment (stars) and under hard frequency floors of 0 to 150 cm^−1^ (circles, light to dark) for every GFN2-xTB pair; the three pairs to the right of the dotted line are at the allylic chloride, and asterisks mark the chain-length pair of Section 3.8. The density functional result, which reverses the majority channel at the saturated dyad, is given in Table 5.

Figure 6 and Table S4–S6 collect what the answer depends on. The variations of Section 2.11 are taken in turn, beginning with the one that changes the answer.

*The level of theory.* The level of theory is the one variation that changes the answer, and it is treated in Section 3.6 and Table 5. At the GFN2-xTB saddle points of all eight pairs, PBE0-D3(BJ)/def2-SVP single points at *ε* = 3.4 give electronic barrier differences of −30 to −49 kJ mol^−1^ at the saturated dyad and −42 to −55 kJ mol^−1^ at the allylic chloride, 13 to 32 kJ mol^−1^ further toward substitution than GFN2-xTB in the two ALPB solvents that bracket this permittivity, furan and diethyl ether (Table S6). The shift has the same sign for every pair, so the shift toward substitution found for the two re-optimized pairs is not specific to them, although, as Section 3.7 and Section 4.5 show, single points alone do not establish the majority channel. On the diborate and boroxol clusters of Section 3.7 single points at the semiempirical geometries point the other way, and re-optimization of the diborate pair at the DFT level points back toward substitution, although Section 3.7 shows that its substitution saddle point is only approximate.

*The dielectric is the dominant semiempirical sensitivity.* The electronic barrier difference $\Delta \Delta E^{‡}$ varies by 30 to 64 kJ mol^−1^ across the twelve ALPB solvents, depending on the pair, as Table S5 lists in full, and it does not vary smoothly with *ε* (Figure 6a). Chloroform lies off the trend of its neighbors for every pair, which is why no single solvent is used as a proxy for PVC. Within the band, the zero-point and entropic terms, 20 to 23 kJ mol^−1^ in favor of elimination at the saturated dyad, exceed the 7 to 12 kJ mol^−1^ spread of the electronic term, and elimination stays in the majority; below the band the selectivity approaches parity or turns over (Section 3.6). A continuum model cannot represent a polymer melt at a filler interphase to better than this spread, which is why the results are given as a band.

*The scan schedule affects the absolute scan barriers but hardly their difference.* Across five schedules (the default schedule of Section 2.4, the leaving group driven to 2.9 and to 3.6 Å, a 51-point grid, and the base lagging the leaving group), the individual barriers span 110.3 to 137.1 kJ mol^−1^, a range of 26.8, while the barrier difference spans 7.5 to 13.1, a range of 5.6. Individual scan barriers should therefore not be read as barrier heights; the difference is far better behaved, which is why differences are reported throughout.

*Conformation and chain length.* Sampling the transition-state conformers changes $\Delta \Delta G^{‡}$ in chloroform by −4.2 to +15.2 kJ mol^−1^ relative to the original pair (Table 4), most for the tridentate graft and the free silanetriol, and moves the band medians by up to 23 percentage points, without changing the majority channel in any case. A single transition-state pair is therefore not a precise estimate of the barrier difference, and the conformer-summed values are the ones used throughout. Recomputing the grafted-silane pair on the tetramer instead of the dyad changes $\Delta \Delta G^{‡}$ from +23.2 (the chain-length dyad pair, a transition-state pair separate from that of Table 4) to +13.4 kJ mol^−1^ (quasi-RRHO, chloroform), a shift of 9.8. Both remain on the elimination side, at more than 99% and 98%, but the dyad is not converged for this quantity, which the ground-state test of Section 3.3 could not detect.

*The entropy treatment and tunneling change the numbers modestly.* Across the quasi-RRHO treatment and the five hard floors, the elimination fraction at the saturated dyad in chloroform stays between 93 and 100%, and no system changes by more than 5.4 percentage points; at the allylic site the treatment matters much more, with fractions from 38 to 99% (Figure 6b, Table S4). Tunneling raises the elimination fraction by up to 7 percentage points and never lowers it.

### 3.9. The Allylic Site: Amplification and a Shift Toward Substitution

**Figure 7:**
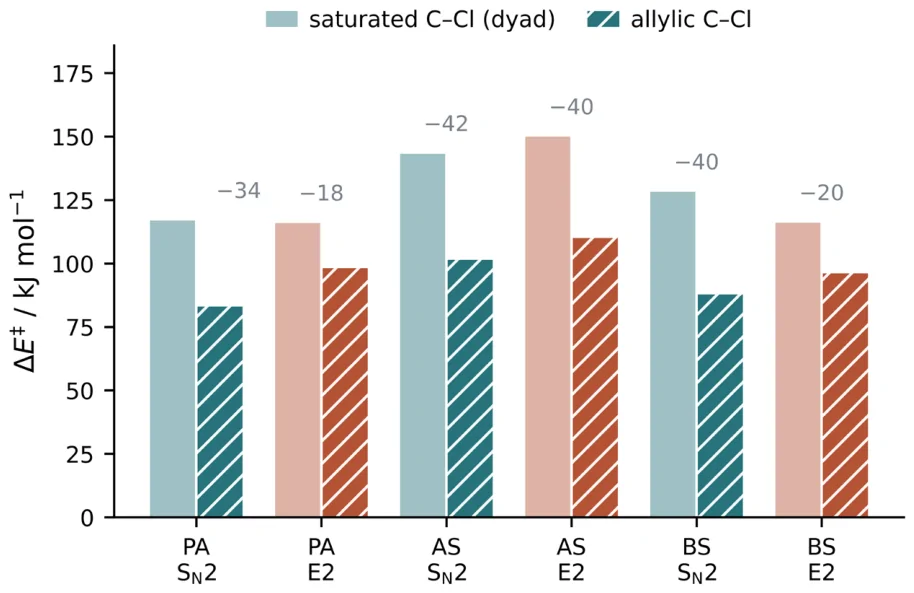
Autocatalytic amplification. GFN2-xTB relaxed-scan barriers (ALPB, chloroform) for substitution (teal) and elimination (rust) at the saturated PVC dyad (pale bars) and at 4-chloro-2-pentene, the allylic chloride produced by one elimination event (hatched bars). PA = *n*-propylamine, AS = 3-aminopropylsilanetriol, BS = B–O–Si bridged grafted silane. Annotations give the barrier change in kJ mol^−1^; every channel is accelerated, and substitution more than elimination, which is the origin of the shift toward substitution in Section 3.9.

If elimination occurs at all, its product matters more than its rate, because the immediate product is an allylic chloride and the autocatalytic character of PVC dehydrochlorination is attributed precisely to the enhanced lability of such structures [23,24]. Repeating the full set of calculations on 4-chloro-2-pentene gives barrier reductions of 17.8 to 41.8 kJ mol^−1^ (Figure 7). For the grafted species substitution falls from 128.2 to 87.8 kJ mol^−1^ and elimination from 116.1 to 96.1 kJ mol^−1^, corresponding at 453 K to rate enhancements of roughly 4.6 × 10^4^ and 2.0 × 10^2^. These are GFN2-xTB scan-level numbers and inherit the caveats of Section 3.8 on absolute barriers; what they establish is the direction and the order of magnitude. At this level the elimination cascade is self-accelerating, as required for the zipper mechanism, and the substitution barrier collapses further still at the allylic site, which is the origin of the shift toward substitution described next.

The preference between the channels also changes. At the allylic chloride the ordering reverses under GFN2-xTB: over the band, elimination accounts for a median of 2% of encounters for the bare aminopropyl model, 6% for the free silanetriol and 9% for the grafted silane, so substitution is the majority channel in all three, with chloroform the only solvent that lies off this trend (Figure 6a). The tethered amine would then form C–N bonds preferentially at sites it has already damaged, so that the coupling that occurs is coupling to a partially conjugated backbone rather than to pristine PVC. Under GFN1-xTB, however, the same three systems give band medians of 67 to 79% elimination, which does not reproduce the reversal. PBE0-D3(BJ) single points at the GFN2-xTB saddle points favor substitution at the allylic chloride by 42 to 55 kJ mol^−1^ in the electronic term (Section 3.8), but these pairs were not re-optimized at that level. The allylic result is therefore reported as the weaker of the two, and the conclusions rest on the density functional result at the saturated backbone unit (Section 3.6).

### 3.10. The Allylic C–Cl Bond Is Weaker

A shielding composite sits in a radiation field, and homolysis of C–Cl bonds is a primary radiolytic event in PVC. This section asks a narrower, thermodynamic question: whether the allylic chloride formed by elimination has a weaker C–Cl bond than the backbone unit it replaces. Whether that translates into faster radiolytic degradation of the composite is a separate question, discussed in Section 4.1.

**Figure 8:**
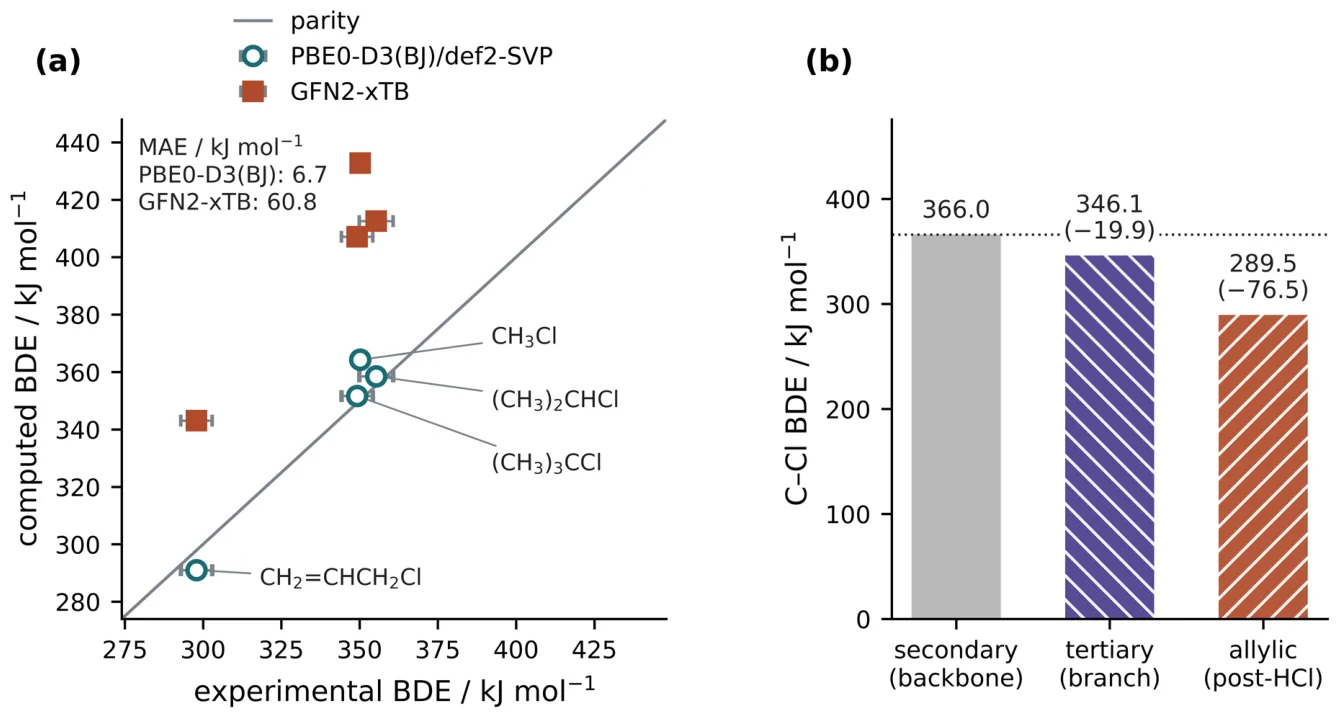
C–Cl bond weakening at the allylic site. (**a**) Validation of the bond-dissociation protocol against the four small chlorides with measured values, with the parity line; horizontal bars are the experimental uncertainties. Experimental bond dissociation enthalpies are assembled from gas-phase enthalpies of formation in the NIST Chemistry WebBook [63] with $\Delta_{f}H(Cl•)$ from [64]; all other values in this figure are computed in this work. PBE0-D3(BJ)/def2-SVP (open teal circles) lies on parity, while GFN2-xTB (filled rust squares) overestimates every bond by 45–83 kJ mol^−1^ and is shown for comparison only. Each chloride is labeled by its formula at its PBE0-D3(BJ) marker; its GFN2-xTB marker lies at the same abscissa. MAE, mean absolute error. (**b**) The three PVC models at PBE0-D3(BJ)/def2-SVP: the normal secondary backbone unit, the tertiary branch-point defect and the allylic chloride formed by one amine-induced elimination. The value above each bar is its bond dissociation energy in kJ mol^−1^, with the change relative to the backbone bond (dotted line) in parentheses. The ordering reproduces the labile-chlorine hierarchy established experimentally for PVC.

The bond dissociation energies of Equation (4) were computed with PBE0-D3(BJ)/def2-SVP, and Table 6 and Figure 8a give its validation. Across four small chlorides with measured bond dissociation enthalpies, PBE0-D3(BJ)/def2-SVP has a mean signed error of +3.2 kJ mol^−1^ and a mean absolute error of 6.7, against a mean signed error of +60.8 for GFN2-xTB, which overestimates every bond in the set by 45 to 83 kJ mol^−1^. The GFN2-xTB values are therefore given for comparison only and no conclusion rests on them.

Applied to the PVC models, that method gives 366.0 kJ mol^−1^ for the secondary backbone C–Cl, 346.1 for the tertiary defect and 289.5 for the allylic chloride (Figure 8b). The allylic bond is therefore 76.5 kJ mol^−1^ weaker than the backbone bond it replaces, and the tertiary defect 19.9 kJ mol^−1^ weaker. GFN2-xTB gives −74.6 and −13.4 kJ mol^−1^ for the same two differences, although its absolute values for the PVC models lie 43 to 49 kJ mol^−1^ above the PBE0-D3(BJ) ones. The ordering, allylic considerably more labile than tertiary and tertiary more labile than normal secondary, reproduces the hierarchy of labile-chlorine structures established experimentally in the PVC degradation literature [21,24,25], an agreement obtained without fitting to it.

### 3.11. A Screening Rule Across the Silane Series

**Figure 9:**
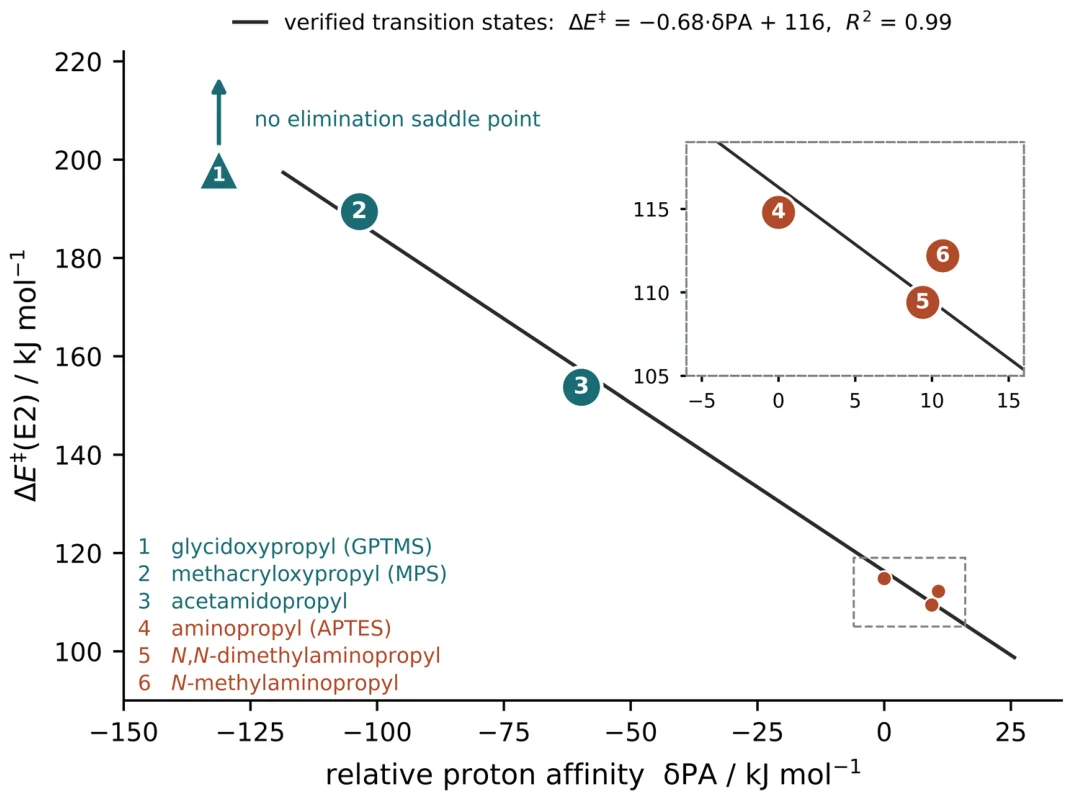
Silane design map. Elimination barrier of the verified transition state, measured from the relaxed encounter complex, against the relative proton affinity descriptor δPA of Equation (3) evaluated on conformer ensembles, for the grafted silane series (GFN2-xTB, ALPB chloroform). Amine-terminated silanes are shown in rust and non-amine silanes in teal; numerals inside the markers key to the list at lower left, and the inset enlarges the three amines. Glycidoxypropyl (triangle) has no elimination saddle point and is placed at its descriptor value only. The line is the fit over the five verified points and serves as a ranking device, not a predictive equation.

The finding that APTES is not inert toward PVC is of limited value without an alternative and a way of choosing one. Eight organofunctional silanes were therefore examined in their grafted form. Two of them, vinyl and *n*-propyl, carry no lone pair, so neither the descriptor nor an elimination path through the organofunctional group is defined for them; they enter the analysis only as the limiting case of a silane that cannot act as a base. For the remaining six, the elimination transition state against the PVC dyad was located and verified as in Section 2.4, and its barrier from the relaxed encounter complex was compared with the descriptor of Equation (3). Five saddle points were verified, with imaginary frequencies between −792 and −1279 cm^−1^. For glycidoxypropyl no elimination saddle point was found: every search returned only a soft torsional mode below 16 cm^−1^ in magnitude with an overlap of at most 0.22 with the reaction coordinate, below the acceptance limit of Section 2.4, because the energy rises without turning over as the epoxide oxygen approaches the β-hydrogen. Results are in Table 7, Figure 9 and Table S9.

The barrier follows the descriptor closely. Over the five verified silanes, $\Delta E^{‡}(E2)$ = −0.68 δPA + 116.3 kJ mol^−1^ with $R^{2}$ = 0.99, and the quasi-RRHO free-energy barrier gives −0.61 δPA + 103.4 kJ mol^−1^, also with $R^{2}$ = 0.99. Leave-one-out refits give slopes between −0.62 and −0.70, and 10,000 bootstrap resamples give a 95% interval of −0.72 to −0.28 for the slope of the electronic barrier; the less negative bound comes from resamples that contain only the three amines, whose descriptors span 11 kJ mol^−1^. With five points and the leverage concentrated in the two carbonyl silanes, the regression is offered as a rank-ordering device, not as a predictive equation, and no barrier is predicted from it anywhere in this paper. What it establishes is the direction and the magnitude: weaker bases give higher elimination barriers, and the difference between a basic and a non-basic organofunctional group is tens of kJ mol^−1^ rather than a few. Three specific findings follow, and one of them contradicts a common expectation.

With respect to the amine-mediated reactions studied here, non-basic organofunctional groups are much safer. Methacryloxypropyl, the organofunctional group of MPS, raises the elimination barrier to 189.4 kJ mol^−1^, 74.6 kJ mol^−1^ above aminopropyl and 67.2 kJ mol^−1^ in free energy, and glycidoxypropyl, that of GPTMS, offers no elimination saddle point at all. Vinyl and *n*-propyl possess no lone pair and offer no elimination channel through the organofunctional group. The substitution channel of these silanes was not computed; none of them carries a nucleophilic nitrogen, so the *N*-alkylation that dominates for the two primary amines treated at the DFT level (Section 3.6) is not available to them. On this preliminary screening evidence the standard non-amine silanes are the appropriate choice for avoiding these amine-mediated reactions in a PVC-matrix shielding composite; their adhesion to $B_{4}C$ and PVC, any reactions of their own with the matrix, their effect on filler dispersion and radiation resistance and their effect on shielding performance were not computed.

Increasing steric bulk at nitrogen does not help against elimination. *N*-methylation and *N*,*N*-dimethylation give electronic barriers of 112.2 and 109.4 kJ mol^−1^, both below the 114.8 kJ mol^−1^ of aminopropyl, and free-energy barriers 3 to 4 kJ mol^−1^ lower. Alkylation raises the basicity (δPA of +10.7 and +9.4 kJ mol^−1^), and that outweighs whatever steric protection it confers. For the elimination channel the intuitive remedy of hindering the amine is therefore counterproductive. The substitution channel, which dominates at the DFT level, was not computed for the alkylated amines; steric hindrance generally slows substitution, so no overall advantage or disadvantage of *N*-alkylation is claimed here.

Amide capping does help, and it falls on the same trend. Acetamidopropyl raises the elimination barrier to 153.7 kJ mol^−1^, 38.9 kJ mol^−1^ above aminopropyl and 34.9 kJ mol^−1^ in free energy. Its relative proton affinity is −59.7 kJ mol^−1^, in line with the lower gas-phase basicity of amides than of amines, so capping works because it removes basicity. Converting a pendant amine to an amide before compounding therefore suppresses the elimination channel computed here; its effect on adhesion, filler dispersion and the performance of the composite was not computed. The descriptor should still be used as a screen, not as a substitute for computing the barrier of a serious candidate.

## 4. Discussion

### 4.1. The Results in the Context of Prior Work

*Substitution versus elimination.* The competition between $S_{N}2$ and E2 has been analyzed in detail for anionic bases, where a stronger base favors elimination and solvation, by weakening the base, shifts the balance toward substitution [78]. A neutral amine behaves differently in one respect that matters here: both of its transition states separate charge on the way to an alkylammonium chloride ion pair, as in the Menshutkin reaction, whose rate depends strongly on the polarity of the medium. The dielectric dependence found in Section 3.8, with polar continua favoring the more charge-separated elimination transition state and low-permittivity continua favoring substitution, is the neutral-base counterpart of that behavior. Experimentally, substitution and elimination are both observed when PVC is treated with an amine [30]. Amines graft onto PVC by substitution at its secondary C–Cl units [79], while ammonia at elevated temperature dechlorinates PVC predominantly by elimination, to polyene sequences and ammonium chloride [80]; the two outcomes are the two channels computed here. For a primary alkylamine at a saturated C–Cl unit, the DFT result of Section 3.6 places the balance on the substitution side, in line with the grafting of amines onto PVC that is observed experimentally.

*The size of the barriers.* Unimolecular HCl elimination from PVC model compounds has computed barriers of 167 to 243 kJ mol^−1^, lowered by an adjacent allyl group [27]. The amine-assisted barriers of this work, 147 to 175 kJ mol^−1^ for substitution and 166 to 186 kJ mol^−1^ for elimination at the DFT level (1 mol L^−1^, 453 K; Table 5), lie at the lower end of or below the unimolecular values, which is what an added base should do, and the allylic activation of Section 3.9 has the same direction as the allyl effect reported there. Thermogravimetry puts the apparent activation energy of the dehydrochlorination step of PVC pyrolysis at 115.7 ± 0.7 kJ mol^−1^ [28] and model-free analysis of chlorinated PVC at 140 kJ mol^−1^ [29]; these are apparent values for a defect-initiated, autocatalytic process and are not directly comparable with a single elementary barrier, but they set the scale on which an amine-assisted pathway has to compete.

*Bond weakening is not yet radiolytic sensitization.* Section 3.10 establishes that the allylic C–Cl bond left by one elimination is 76.5 kJ mol^−1^ weaker than the backbone bond. That is a thermodynamic precondition for preferential homolysis, not a demonstration that the composite degrades faster under irradiation. Radiolysis of PVC proceeds through excitation and ionization of the whole matrix, and which bonds finally break is decided by charge and exciton migration and trapping, radical diffusion, the presence of oxygen, dose rate, humidity and temperature [34,35,37]. A filler surface adds its own charge traps and its own secondary electrons. Whether the weaker allylic bonds made at the interphase are cleaved preferentially under these conditions can only be settled by the irradiation experiment proposed in Section 4.4; the calculation identifies the defect and its bond strength, and nothing more.

### 4.2. Filler Loading and Matrix Stability Are Coupled Through the Interphase

A grafted aminosilane that reacts consumes C–Cl units of the interphase and leaves an allylic chloride wherever it eliminates, in proportion to grafting density and hence to filler specific surface area. The scaling is what makes this a formulation problem rather than a curiosity. The purpose of raising $B_{4}C$ loading is to increase neutron capture, and the purpose of reducing particle size is to improve dispersion and to reduce the self-shielding of individual grains, in which the interior of a particle is screened by its own surface and contributes less capture than its mass implies. Both moves increase interfacial area per unit volume of composite, and with it the number of grafted amines and the number of labile chlorides they can form. A nanoscale or submicron filler, which is the direction the shielding-composite literature has been moving in, therefore maximizes the quantity this mechanism is proportional to. Aminosilanes are used for exactly this purpose: APTES has been applied to $B_{4}C$ in polyimide shielding composites [4], and in a PVC matrix the same choice would introduce the chemistry described here.

Two things soften the trade without removing it. Under the preliminary estimate of Section 3.2, a PVC matrix reaches the absorption cross section of high-density polyethylene carrying 10 wt% $B_{4}C$ with 6.2 wt% $B_{4}C$, so the matrix substitution that motivates this study also reduces the interfacial area the composite carries. And the damage per grafted amine is bounded: the immediate product of either channel is an alkylammonium chloride, and the nitrogen is no longer a base unless the HCl escapes the melt or is taken up by a neighbor. What is not bounded is the consequence, because the allylic chloride that one elimination leaves behind is a defect the polymer can amplify on its own, by the zipper mechanism [23].

*N*-alkylation is not only a loss. It forms a covalent bond between the grafted silane and the matrix, which is the link a coupling agent is meant to supply, at the cost of an alkylammonium chloride at the interface and, in a minor fraction of encounters, an allylic chloride. Whether the net effect on adhesion and durability is positive is not addressed by these calculations; it is part of what the experiment of Section 4.4 would decide.

### 4.3. Choosing a Different Coupling Agent

APTES is not inert toward PVC, and that finding is of limited value without an alternative. Section 3.11 supplies a preliminary screen, and the ranking it gives is more useful than the regression behind it.

The methacryloxypropyl group raises the elimination barrier by 74.6 kJ mol^−1^ relative to aminopropyl, and the glycidoxypropyl group offers no elimination saddle point at all. Methacryloxypropyl and glycidoxypropyl silanes are standard commercial products, are already used with other matrices, and require no change to compounding practice. On this screening evidence they are the candidates to test first in a PVC-matrix shielding composite. The recommendation is limited to the amine-mediated substitution and elimination pathways computed here; it does not establish that these silanes are unreactive toward PVC by other routes, or that they give better adhesion, filler dispersion, mechanical performance, radiation resistance, shielding or overall composite performance, which have to be measured experimentally.

Where an amine must be retained, for instance because the same coupling agent has to serve a second function, amide capping is the effective modification in this screen: the acetamidopropyl group raises the elimination barrier by 38.9 kJ mol^−1^. This result, too, concerns only the amine-mediated elimination pathway computed here and not the performance of the composite. Computed from conformer ensembles, its relative proton affinity is −59.7 kJ mol^−1^, and it falls on the same trend as the other silanes (Section 3.11).

The intuitive remedy of hindering the amine sterically does not work against elimination. *N*-methylation and *N*,*N*-dimethylation lower the elimination barrier rather than raising it, because the increase in basicity outweighs whatever steric protection the alkyl groups confer. Their effect on substitution, the channel that dominates at the DFT level, was not computed, and hindered amines are therefore not recommended either way.

### 4.4. An Experimental Test

The central claim is falsifiable and the experiment is straightforward. Filler, loading, particle size distribution and compounding history are held constant and only the silane varied, across three arms: $B_{4}C$ treated with APTES, $B_{4}C$ treated with a non-basic silane of comparable chain length, and untreated $B_{4}C$. Grafting density must be measured rather than assumed, by thermogravimetry or elemental analysis, since the predictions concern reactions per grafted amine.

Four observables discriminate between the channels. XPS of the interfacial region measures substitution directly, through the growth of a substituted-nitrogen component in the N 1s envelope, following the approach applied to bulk PVC amination by Park et al. [30]. Elimination is measured by its products: polyene sequences absorb progressively further into the visible as they lengthen, quantifiable by diffuse-reflectance UV–visible spectroscopy; the conjugated C=C Raman bands near 1120 and 1510 cm^−1^ report the polyene population and sequence length directly; and evolved HCl during a controlled thermal ramp, by conductometric titration of the effluent gas, should show a shortened induction time if allylic chlorides have been formed. The semiempirical and density functional descriptions predict very different ratios of nitrogen incorporation to polyene formation, so that ratio is itself the discriminating quantity.

One prediction concerns the mechanism of elimination rather than its share. The kinetic isotope effect was computed with all β-hydrogens of the PVC model replaced by deuterium (Table S8). At the GFN2-xTB level $k_{H}/k_{D}$ is 2.9 to 3.0 for elimination at 453 K for all three amine models (3.5 to 3.6 with the Wigner tunneling factor), against 0.89 to 0.94 for substitution, a small inverse secondary effect. For *n*-propylamine, β-deuteration therefore changes the elimination/substitution branching ratio by a factor of 3.1 in the synchronous semiempirical description and, from the transition-state Hessians alone, by a factor of 2.3 in the asynchronous DFT description, in which the transferred proton still loses much of its C–H zero-point energy; for the grafted silane the two factors are 3.3 and 1.5. Both descriptions predict a primary isotope effect on HCl evolution with no corresponding decrease in nitrogen incorporation. The isotope effect would confirm that elimination proceeds by β-C–H cleavage; the ratio of nitrogen incorporation to polyene formation is what separates the two descriptions.

The radiation-specific test follows from Section 3.10 and Section 4.1. Specimens from each arm, irradiated to matched dose and then compared for HCl yield and polyene absorption, should show the aminosilane arm degrading faster per unit dose if the allylic chlorides it generates are cleaved preferentially. Because grafted amines are few compared with the C–Cl units and the labile chlorines already present in commercial PVC, the interfacial signal may be small, and a high-surface-area filler or an interphase-sensitive probe will be needed to resolve it. That comparison is the direct test, and the dose-rate and humidity dependences documented for unfilled PVC [34,35] provide the necessary controls.

### 4.5. Which Conclusions Are Robust, and Limitations

Table 8 sorts the results by the tests that were applied to them. Two results pass every test applied: an elimination event leaves an allylic C–Cl bond 76.5 kJ mol^−1^ weaker than the backbone bond, and non-basic organofunctional groups raise the elimination barrier by tens of kJ mol^−1^. Whether an amine reacts with the polymer during processing depends on its state: a free amine does so at both levels of theory, but for the grafted silane the density functional half-life at 453 K, hours to days for a single conformer, and only an order-of-magnitude estimate for an idealized bimolecular encounter, reaches processing times only at the lower end of its uncertainty (Section 3.6). The identity of the majority channel at a saturated unit is the one result that density functional theory reverses: the two semiempirical Hamiltonians agree with each other and with every internal test within the dielectric band, and density functional theory reverses them. The conclusions of this work follow the DFT result for the two principal pairs. The larger interface models do not settle the question: at the GFN2-xTB level they favor elimination, and the only one re-optimized at the DFT level, the diborate, favors substitution, with the approximate substitution saddle point described in Section 3.7. The reversal of selectivity at the allylic chloride is model-dependent and is not relied on.

Sorted by the level of theory that supports them, the results fall into three groups. Supported at the DFT level are the asynchronous character of the elimination transition state, the reduced zero-point advantage of elimination, the preference for substitution for *n*-propylamine and the monodentate graft on the single boron site, the absolute barriers and half-lives of these two pairs, and the weakening of the allylic C–Cl bond. Indicated at the DFT level, with an approximate substitution saddle point, is the same preference on the diborate. Supported only at the semiempirical level, or at the DFT level by single points alone, are the conformer-summed selectivities, the dielectric and entropy dependences, the effect of the attachment mode, the results on the boroxol ring and with explicit water, the selectivity at the allylic chloride and the silane series. At the saturated dyad and on the diborate, where both levels were applied with re-optimized transition states, they disagree on the majority channel, so the semiempirical results on models without such a DFT counterpart establish trends within that method and not the majority channel.

Beyond the level of theory, which Section 3.6 shows to be decisive, the dielectric environment is the largest single uncertainty in the electronic term. A continuum cannot represent a polymer melt at a filler interphase, in which the local environment of a reacting pair is neither uniform nor isotropic, and the band of Section 2.6 states that uncertainty; below a permittivity of about 2.9 the selectivity changes. The zero-point and entropic terms were computed at the chloroform geometries only and carried across the band unchanged.

The density functional validation has its own limits. It covers two of the eight pairs and the diborate model, at one permittivity (3.4) and with one conformer per channel; the conformer sampling at the GFN2-xTB level changed the barrier difference by up to 15 kJ mol^−1^ across the eight pairs, although only by +0.9 and −4.2 kJ mol^−1^ for the two pairs validated here, against a DFT free-energy preference for substitution of 12 to 19 kJ mol^−1^. The analytic Hessian with the continuum did not fit in the available memory, so the saddle points were verified in a subspace of the softest modes and the thermal term was taken from a Hessian whose reacting-core block is DFT and whose remainder is GFN2-xTB. Besides the reaction mode, this mixed Hessian has two or three soft negative eigenvalues, at most 120 cm^−1^ in magnitude (134 cm^−1^ on the diborate), which arise from joining the two levels of theory and enter the thermal term by magnitude; setting them instead to 50, 100 or 150 cm^−1^, or omitting them, changes the thermal term by at most 2.5 kJ mol^−1^ (3.8 kJ mol^−1^ on the diborate) and the elimination fraction to at most 6%. The DFT saddle points were verified by their curvature in a subspace, and the connectivity of the four saddle points of the two principal pairs was checked by downhill relaxations in both directions rather than by full intrinsic reaction coordinate calculations. The relaxations lead from each of them to the reactant side and to the product side, as Table S11 lists, but they were stopped before full convergence and do not trace the path between them. The diborate pair was not tested in this way, and no coupled-cluster reference was computed. Together these limits make the DFT branching fraction an estimate of order of magnitude rather than a precise number, and not a product ratio for a real composite, in which conformational freedom, local polarity and access to C–Cl units differ from those of the model pairs. The surface model enters as well: on the diborate of Section 3.7, density functional single points at the semiempirical geometries shifted the electronic barrier difference by 28 to 51 kJ mol^−1^ toward elimination, but re-optimization at the DFT level restored the preference for substitution (−40 kJ mol^−1^ in electronic energy and −31 kJ mol^−1^ in $\Delta \Delta G^{‡}$ at 453 K, with an approximate substitution saddle point) through a hydrogen bond from the cluster to the leaving chloride. With one transition-state conformer per channel, the hydrogen-bond pattern at a real, oxide-rich surface remains an uncertainty of this size. The two levels of theory disagree on the one larger interface model that both treated with re-optimized transition states: GFN2-xTB favors elimination on the diborate, as it does on the boroxol ring and with explicit water, whereas DFT favors substitution on the diborate. This is evidence that the computed selectivity at the interface is method-dependent, and it limits what can be claimed for the interface: the DFT preference for substitution is supported for the single boron site and indicated for the diborate, and it is not extended here to the boroxol ring, to an oxide overlayer or to other $B_{4}C$ surface environments, none of which was treated with re-optimized transition states at the DFT level.

The models are molecular. The larger clusters and explicit water of Section 3.7 test the local interface chemistry, but they do not carry surface constraint or cooperativity at realistic grafting densities. The PVC dyad captures the local electronic environment of a C–Cl unit but not chain entanglement, free-volume constraints on the approach geometry, or the reduced conformational freedom of a tethered reactant; the tetramer test of Section 3.8 shows that chain length changes the barrier difference without changing its sign. Concerted E2 elimination requires an anti-periplanar β-hydrogen, and a chain segment pinned against a rigid ceramic surface cannot always supply one, so the effective elimination rate in a real composite will be lower than the model implies by some accessibility factor. Substitution is subject to an analogous but distinct constraint, and there is no reason to assume the two cancel.

A periodic transition-state search, with the grafted silane and the PVC model on the hydroxylated slab, was not attempted. It requires a method that treats the organic tether and the ceramic on the same footing with reliable second derivatives, and neither the foundation potential used for the solid, which was not trained on molecular organic chemistry, nor a tight-binding treatment of a slab of this size is adequate for free-energy differences of the size computed here.

The surface model is one termination of one composition. $B_{4}C(0001)$ was chosen because it is the plane the hexagonal cell cleaves along and because the cut can be made with few broken bonds, not because it is known to dominate a real powder; $\left(10\bar{1}1\right)$ is the surface studied most carefully from first principles [72]. Real boron carbide powder carries an oxidized surface layer [75], so the site an approaching silanol meets may be a boron-oxide overlayer rather than a hydroxylated carbide plane, which is why the boroxol-ring cluster of Section 3.7 was included. The hydroxyl coverage of the slab is the ideal fully hydroxylated limit and overstates what a real powder carries, and a nanoscale powder, with its higher curvature and higher defect density, will depart from it further still.

The shielding estimate is preliminary and transport-free (Section 2.10). It gives capture per unit path length and hydrogen density, not a shielding performance: self-shielding within filler particles, the angular and energy distribution of a real source, secondary capture gammas from both chlorine and boron, porosity and the mechanical consequences of high filler loading are all outside it.

Finally, no ionizing radiation was simulated. Section 3.10 establishes a difference in bond strength, not a radiolysis calculation; as Section 4.1 sets out, whether that difference produces faster radiolytic degradation is an experimental question.

## 5. Conclusions

This work examined computationally whether an aminosilane coupling agent grafted on boron carbide reacts with a poly(vinyl chloride) matrix, in the context of a neutron-shielding composite. The substrate was built and validated rather than assumed: a periodic $B_{4}C$ cell reproduces the measured lattice parameters to 0.09% in *a* and 1.30% in *c*, and its hydroxylated (0001) surface presents a boron–oxygen bond within 0.01 Å of that of the molecular cluster used for the interface chemistry.

Under a preliminary, transport-free estimate, 6.2 wt% $B_{4}C$ in PVC gives the same macroscopic thermal-neutron absorption cross section as 10 wt% in high-density polyethylene, because the chlorine of PVC is itself a thermal absorber. Less filler means less interfacial area and less coupling agent. Grafting is thermodynamically accessible: APTES hydrolysis is exergonic by 33.4 kJ mol^−1^ overall, and condensation onto a boron hydroxyl site by 15.6 kJ mol^−1^.

At a saturated backbone C–Cl unit, the semiempirical GFN2-xTB and GFN1-xTB methods make elimination of HCl the majority channel for every amine model and attachment mode, because in their synchronous elimination transition state the hydrogen in flight removes a high-frequency C–H stretch from the zero-point sum; averaged over ten pairs, this zero-point and thermal term favors elimination by 12 kJ mol^−1^ and entropy by a further 6 kJ mol^−1^. Dispersion-corrected hybrid density functional theory, applied to the free amine and to the grafted silane, does not confirm this preference. Its elimination transition state is asynchronous, with the proton already on the nitrogen, the zero-point advantage shrinks to 7 kJ mol^−1^, and substitution is favored by 12 to 19 kJ mol^−1^ in free energy, for one transition-state conformer per channel and with thermal terms from mixed DFT/GFN2-xTB Hessians. At the level of theory on which the conclusions rest, the amine therefore reacts with PVC mainly by substitution, forming a C–N bond to the polymer and converting itself to the hydrochloride, and eliminates HCl in only a small fraction of encounters. These results show that the semiempirical prediction of dominant elimination is not reliable; they do not give a precise product ratio for a real composite. On the diborate, the only larger cluster re-optimized at the DFT level, the semiempirical and density functional methods disagree, and the semiempirical preference for elimination on the other larger models has no DFT re-optimization behind it. Because the diborate substitution saddle point is only approximate, the preference for substitution is supported at the DFT level for *n*-propylamine and for the graft on the single boron site, indicated for the diborate, and not established for other $B_{4}C$ surface environments. The same level gives a free amine a half-life of minutes in the melt at 453 K, but a grafted amine one of hours to days for a single transition-state conformer, both order-of-magnitude estimates for an idealized bimolecular encounter, so that only part of the grafted amines can be expected to react during processing; semiempirical screens of amine–alkyl chloride selectivity should be checked against such a calculation.

At the allylic chloride left by one elimination, both channels are faster, and under GFN2-xTB substitution becomes the majority channel, so that C–N bonds would form preferentially at chain positions the amine has already damaged; GFN1-xTB does not reproduce this reversal, and the result is model-dependent. The allylic C–Cl bond is 76.5 kJ mol^−1^ weaker than the backbone bond it replaces at PBE0-D3(BJ)/def2-SVP, a method validated to a mean absolute error of 6.7 kJ mol^−1^ against four measured bond dissociation enthalpies. Whether these weaker bonds are cleaved preferentially under irradiation is not established by the calculations and requires the irradiation experiment proposed in Section 4.4.

Across eight grafted silanes, six of which carry a basic site, the elimination barrier falls as the basicity of the organofunctional group rises, closely enough to rank candidates but not to predict barriers. The methacryloxypropyl group raises the barrier by 75 kJ mol^−1^ and the glycidoxypropyl group offers no elimination saddle point, so these standard non-basic silanes are the candidates to test first in a PVC-matrix shielding composite, as far as the amine-mediated reactions studied here are concerned; their adhesion, effect on filler dispersion, radiation resistance and shielding performance were not assessed and require experimental validation. *N*-alkylation of the amine lowers the elimination barrier, whereas amide capping raises it by 39 kJ mol^−1^; the substitution barrier was not computed across the series, and this result, like the ranking above, concerns only the amine-mediated pathways computed here.

For radiation shielding, the practical implication is that filler loading and matrix chemistry are coupled through the interphase. Raising the $B_{4}C$ content to increase neutron capture, or reducing the particle size to improve dispersion and limit self-shielding, increases the interfacial area and hence the amount of grafted silane. If that silane is an aminosilane, grafted amines can be consumed by the matrix, slowly at processing temperature, mostly by substitution to the hydrochloride and in a minor fraction by elimination, which leaves a weakened allylic C–Cl bond in the matrix. The effect is partly offset by the lower filler loading a PVC matrix needs, and this amine-mediated chemistry can be avoided by choosing a non-basic coupling agent. Measuring the partition between nitrogen incorporation and HCl evolution would decide between the semiempirical and the density functional description, and its kinetic isotope effect on β-deuterated PVC would confirm that elimination proceeds by β-C–H cleavage.

## Figures and Tables

**Table 1:** Periodic boron carbide: relaxed cell parameters and geometry against experiment. Both cells were built in the hexagonal setting of $R\bar{3}m$ from the refined coordinates of Kolel-Veetil et al. [65] and relaxed, positions and cell together, with MACE-MP-0. $B_{13}C_{2}$ is ordered in $R\bar{3}m$; $B_{4}C$ carries one polar icosahedral B→C substitution per icosahedron and is not. Deviations are relative to the experimental cell used to build both. The atom count is a property of the construction and not a measurement. The energies per atom belong to different compositions and are not comparable with one another; they are given so that the relaxations can be reproduced. A dash (—) marks quantities that have no experimental counterpart.

Quantity	$B_{13}C_{2}$	$B_{4}C$	Experiment
*a*/Å	5.6333 (+0.64%)	5.5926 (−0.09%)	5.5974
*c*/Å	12.2334 (+1.34%)	12.2280 (+1.30%)	12.0715
*α*, *β*/°	90.00, 90.00	91.03, 88.97	90, 90
*γ*/°	120.00	120.64	120
atoms per cell	45	45	—
shortest contact/Å	1.424	1.400	—
chain C–B/Å; C–B–C/°	1.424; 180.0	1.443; 179.4	1.43; 180
energy/eV atom^−1^	−7.091	−7.181	—

**Table 2:** The boron–oxygen bond in three environments. PBE0-D3(BJ)/def2-TZVP on the isolated $B(OH{)}_{3}$ molecule is the reference. The offset column is each cheap method minus that reference on the same molecule. The surface value is the mean over the seven hydroxyls of the relaxed $B_{4}C(0001)$ slab; correcting it by the MACE-minus-GFN2 offset of +0.022 Å places it on the same footing as the cluster values immediately below it. Values in Å. A dash (—) marks rows for which no offset is defined, because the offset compares two methods on the same isolated molecule.

Species	Method	B–O	Offset vs. PBE0
$B(OH{)}_{3}$, isolated	PBE0-D3(BJ)/def2-TZVP	1.368	—
$B(OH{)}_{3}$, isolated	GFN2-xTB	1.360	−0.008
$B(OH{)}_{3}$, isolated	MACE-MP-0	1.382	+0.014
$B_{4}C(0001)-OH$, slab	MACE-MP-0	1.371 ± 0.002	—
$B_{4}C(0001)-OH$, slab	corrected to a GFN2 footing	1.349	—
$(HO{)}_{2}B-O-Si$ bridge, B–OH	GFN2-xTB	1.358	—
$(HO{)}_{2}B-O-Si$ bridge, B–O–Si	GFN2-xTB	1.353	—

**Table 3:** Molecular species and their GFN2-xTB global-minimum properties. Conformer spread is the energy range of the optimized conformer set; *N*(imag) is the number of imaginary vibrational modes below −10 cm^−1^ after removal of the translational and rotational modes. A dash (—) marks species with a single conformer, for which no spread is defined.

Species	Formula	*N*(atoms)	Conformers	Spread/kJ mol^−1^	HOMO–LUMO/eV	*N*(imag)
PVC hexamer, *RSSRSR*	$C_{13}H_{22}{Cl}_{6}$	41	12	16.7	6.23	0
PVC hexamer, *RRSSRS*	$C_{13}H_{22}{Cl}_{6}$	41	12	17.7	6.39	0
PVC hexamer, *RSRSRS*	$C_{13}H_{22}{Cl}_{6}$	41	12	9.7	6.24	0
PVC tetramer, *RSSR*	$C_{9}H_{16}{Cl}_{4}$	29	12	10.7	6.44	0
PVC dyad (2,4-dichloropentane)	$C_{5}H_{10}{Cl}_{2}$	17	6	15.4	6.73	0
Allylic chloride (4-chloro-2-pentene)	$C_{5}H_{9}Cl$	15	3	5.0	4.86	0
APTES (parent)	$C_{9}H_{23}NO_{3}Si$	37	10	15.4	6.69	0
APTES, monohydrolyzed	$C_{7}H_{19}NO_{3}Si$	31	10	18.2	7.74	0
APTES, dihydrolyzed	$C_{5}H_{15}NO_{3}Si$	25	10	21.4	7.99	0
3-Aminopropylsilanetriol	$C_{3}H_{11}NO_{3}Si$	19	7	16.5	8.14	0
B–O–Si bridged silane, $T^{1}$	$C_{3}H_{12}BNO_{5}Si$	23	10	24.5	6.30	0
*n*-Propylamine	$C_{3}H_{9}N$	13	2	1.7	11.31	0
Boric acid	$H_{3}BO_{3}$	7	1	—	9.10	0
Water	$H_{2}O$	3	1	—	14.39	0
Ethanol	$C_{2}H_{6}O$	9	1	—	12.89	0
Hydrogen chloride	HCl	2	1	—	9.18	0

**Table 4:** Barrier differences between the two channels, kJ mol^−1^, GFN2-xTB in chloroform; the density functional validation is in Table 5. $\Delta \Delta X^{‡}$ is defined by Equation (1); positive favors elimination. $\Delta \Delta H^{‡}$ and $\Delta \Delta G^{‡}$ use the quasi-RRHO treatment at 453 K (Section 2.5); $\Delta \Delta G^{‡}(ens)$ is the conformer-summed value of Equation (2) over the transition-state conformers of Section 2.4. $\Delta \Delta E^{‡}(scan)$ is the quantity obtainable from the relaxed-scan maxima with each barrier measured from its own encounter complex, which, as shown above, is not a well-defined comparison. The remaining columns are differences between verified transition-state energies and need no reference state. PA = *n*-propylamine, AS = 3-aminopropylsilanetriol, BS = B–O–Si bridged grafted silane in its monodentate ($T^{1}$), bidentate ($T^{2}$) and tridentate ($T^{3}$) forms. $\nu^{‡}$ is the imaginary frequency of each transition state. Every structure in this table has exactly one imaginary mode; the two structures that carry a second, soft one are the chain-length pair of Section 3.8 and do not appear here.

Amine	Substrate	$\Delta \Delta E^{‡}(scan)$	$\Delta \Delta E^{‡}$	$\Delta \Delta H^{‡}$	$\Delta \Delta G^{‡}$	$\Delta \Delta G^{‡}(ens)$	$\nu^{‡}(S_{N}2)$/cm^−1^	$\nu^{‡}(E2)$/cm^−1^
PA	PVC dyad	+1.0	−6.43	+6.41	+16.58	+17.46	−522	−1189
AS	PVC dyad	−6.7	−7.99	+4.47	+11.61	+20.93	−534	−1177
$\mathrm{BS}\;(T^{1}$)	PVC dyad	+12.1	+2.75	+15.24	+23.97	+19.76	−537	−1251
$\mathrm{BS}\;(T^{2}$)	PVC dyad	−4.3	−7.47	+5.23	+12.45	+14.87	−518	−1176
$\mathrm{BS}\;(T^{3}$)	PVC dyad	+25.8	−11.23	+2.16	+9.48	+24.67	−533	−1207
PA	Allylic chloride	−15.1	−18.71	−6.56	+2.99	+2.04	−417	−1036
AS	Allylic chloride	−8.6	−12.36	−0.82	+5.17	+4.54	−410	−962
$\mathrm{BS}\;(T^{1}$)	Allylic chloride	−8.3	−12.06	+0.01	+6.70	+4.86	−420	−1049

**Table 5:** Validation at the DFT level: the two pairs re-optimized at PBE0-D3(BJ)/def2-SVP/IEF-PCM(*ε* = 3.4), compared with GFN2-xTB (chloroform, original transition-state pair). The first row gives $\Delta \Delta E^{‡}$ at the level each pair was optimized at: GFN2-xTB or PBE0-D3(BJ)/def2-SVP. $\Delta \Delta X^{‡}$ is defined by Equation (1), in kJ mol^−1^; negative values favor substitution. Energies with def2-TZVP and with the other functionals are single points at the PBE0-D3(BJ)/def2-SVP geometries. The thermal term ($\Delta \Delta G^{‡}$ − $\Delta \Delta E^{‡}$, quasi-RRHO, 453 K) at the DFT level uses a Hessian whose reacting-core block is computed with PBE0-D3(BJ) and whose remainder is taken from GFN2-xTB (Section 2.1). Barriers from the separated reactants are for 1 mol L^−1^ and 453 K: the GFN2-xTB values are conformer-summed medians over the dielectric band, with the band range in parentheses (Section 3.6); the DFT values are for one transition-state conformer per channel, with def2-SVP and def2-TZVP energies, in that order, and mixed-Hessian thermal terms for reactants and transition states alike (Section 2.1). The half-life combines both channels at a C–Cl concentration of 22.4 mol L^−1^. PA = *n*-propylamine; BS = monodentate ($T^{1}$) grafted silane. The elimination percentages are for the original transition-state pair, in chloroform for GFN2-xTB and at *ε* = 3.4 for DFT. Chloroform lies off the trend of the other ALPB solvents (Section 3.8); in furan and diethyl ether, which bracket *ε* = 3.4, the GFN2-xTB $\Delta \Delta E^{‡}$ is −15.5 and −17.3 kJ mol^−1^ for PA and −6.8 and −7.8 kJ mol^−1^ for BS. A dash (—) marks combinations that were not computed: the def2-TZVP and other-functional energies were evaluated at the DFT geometries only. As Section 3.6 and Section 4.5 explain, the DFT elimination percentages and half-lives are order-of-magnitude estimates.

Quantity	PA, GFN2-xTB	PA, DFT	BS, GFN2-xTB	BS, DFT
$\Delta \Delta E^{‡}$, optimization level	−6.4	−27.7	+2.8	−26.4
$\Delta \Delta E^{‡}$, PBE0-D3(BJ)/def2-TZVP	—	−23.7	—	−19.1
$\Delta \Delta E^{‡}$, B3LYP-D3(BJ)/def2-TZVP	—	−23.7	—	−20.7
$\Delta \Delta E^{‡}$, M06-2X-D3(0)/def2-TZVP	—	−28.8	—	−30.5
Zero-point term	+16.4	+7.2	+16.0	+7.0
Thermal and entropic term	+23.0	+8.7	+21.2	+7.2
$\Delta \Delta G^{‡}$ (def2-TZVP energies for DFT)	+16.6	−15.0	+24.0	−11.8
Elimination/% of encounters	99	2	>99	4
$\Delta G^{‡}(S_{N}2)$ from the separated reactants	154 (146–157)	147; 156	167 (159–171)	159; 175
$\Delta G^{‡}(E2)$ from the separated reactants	144 (128–148)	166; 171	154 (139–161)	179; 186
Half-life of the amine at 453 K, both channels	2 min (2 s–5 min)	5 min; 43 min	32 min (33 s–3.2 h)	2.0 h; 4.4 d
$\nu^{‡}(E2)$/cm^−1^	−1189	−128	−1251	−125
$\nu^{‡}(S_{N}2)$/cm^−1^	−522	−426	−537	−444
N···H; C···H in the E2 transition state/Å	1.30; 1.38	1.14; 1.65	1.30; 1.39	1.13; 1.72

**Table 6:** Homolytic C–Cl bond dissociation energies, kJ mol^−1^. Experimental values are $BDE=\Delta_{f}H\left(R•\right)+\Delta_{f}H\left(Cl•\right)-\Delta_{f}H\left(R-Cl\right)$ with $\Delta_{f}H(Cl•)$ = 121.30 kJ mol^−1^ [64]; the enthalpies of formation are from the NIST Chemistry WebBook [63] and the uncertainty is propagated from the uncertainties of the enthalpies of formation of R–Cl and R•. The four small chlorides are the validation set; the three PVC models are computed with the same protocol and no correction is transferred between them. A dash (—) marks entries that do not exist: no measured bond dissociation enthalpy is available for the PVC models, so no error can be given for them, and the two summary rows have values in the error columns only.

Molecule	Experimental	GFN2-xTB	Error	PBE0-D3(BJ)/def2-SVP	Error
Chloromethane	350.2 ± 1.8	432.8	+82.6	364.3	+14.1
2-Chloropropane	355.3 ± 5.4	412.6	+57.3	358.5	+3.2
2-Chloro-2-methylpropane	349.2 ± 5.0	407.1	+57.9	351.7	+2.5
3-Chloro-1-propene	297.9 ± 5.0	343.1	+45.2	291.0	−6.9
mean signed error	—	—	+60.8	—	+3.2
mean absolute error	—	—	60.8	—	6.7
PVC dyad, secondary	—	408.7	—	366.0	—
tertiary defect	—	395.3	—	346.1	—
allylic chloride	—	334.1	—	289.5	—
allylic minus secondary	—	−74.6	—	−76.5	—

**Table 7:** Basicity descriptor and elimination barrier across the grafted silane series, $R-Si(OH{)}_{2}-O-B(OH{)}_{2}$, reacting with the PVC dyad (GFN2-xTB, ALPB chloroform). δPA is defined by Equation (3), evaluated on conformer ensembles of the neutral and protonated forms, and referenced to the aminopropyl case; a negative value denotes a weaker base. Barriers are measured from the relaxed encounter complex to the verified elimination transition state; $\Delta G^{‡}$ uses the quasi-RRHO treatment at 453 K, and $\nu^{‡}$ is the imaginary frequency. GPTMS = 3-glycidoxypropyltrimethoxysilane; MPS = 3-methacryloxypropyltrimethoxysilane. Vinyl and *n*-propyl possess no lone pair, so neither a descriptor nor a reaction path is defined for them. A dash (—) marks these undefined entries, and those of glycidoxypropyl, which has no elimination saddle point. The transition states of *N*-methylaminopropyl, acetamidopropyl and methacryloxypropyl carry a soft second mode below 60 cm^−1^ in magnitude, of the hindered-rotor kind, and the relaxed encounter complexes of *N*-methylaminopropyl and methacryloxypropyl retain one and two soft modes of −15 to −18 cm^−1^; all of these are treated as low-frequency vibrations.

Grafted Silane	Basic Site	δPA/kJ mol^−1^	$\Delta E^{‡}(E2)$/kJ mol^−1^	$\Delta G^{‡}(E2)$/kJ mol^−1^	$\nu^{‡}$/cm^−1^
Glycidoxypropyl (GPTMS)	epoxide O	−131.2	no saddle point	—	—
Methacryloxypropyl (MPS)	ester carbonyl O	−103.5	189.4	169.1	−792
Acetamidopropyl	amide carbonyl O	−59.7	153.7	136.8	−991
Aminopropyl (APTES)	primary amine N	0.0	114.8	101.9	−1191
*N*-Methylaminopropyl	secondary amine N	+10.7	112.2	98.0	−1146
*N*,*N*-Dimethylaminopropyl	tertiary amine N	+9.4	109.4	99.0	−1279
Vinyl	none (alkene π)	—	—	—	—
*n*-Propyl	none	—	—	—	—

**Table 8:** Which results are robust. Each entry states whether the result survives the variation named in the column: GFN1/GFN2, the two semiempirical Hamiltonians; DFT, the PBE0-D3(BJ) validation of Section 3.6; dielectric, the ALPB band of Section 2.6; entropy, the quasi-RRHO treatment and the five frequency floors; conformers, the transition-state conformer ensembles; model size, the tetramer, the larger boron–oxygen clusters and explicit water. For the allylic chloride, the dielectric entry excludes chloroform, which lies off the trend of the other solvents. A dash (—) marks a variation that was not tested or does not apply to the result.

Result	GFN1/GFN2	DFT	Dielectric	Entropy	Conformers	Model Size
Elimination is the majority channel at a saturated C–Cl unit	yes	no	yes (*ε* ≥ 2.9)	yes	yes	GFN2: yes; DFT, diborate: no
Substitution is the majority channel at a saturated C–Cl unit	no	yes	*ε* = 3.4 only	quasi-RRHO only	—	DFT, diborate: yes (approximate $S_{N}2$ saddle point); GFN2, larger clusters: no
The amine reacts with the C–Cl unit during processing	GFN2 only: yes	free amine: yes; graft: uncertain	yes	yes	yes	—
The allylic C–Cl bond is weaker than the backbone bond	GFN2: yes; GFN1: —	yes	—	—	—	—
Substitution is preferred at the allylic chloride	no	$\Delta \Delta E^{‡}$ only (single points)	yes	no	yes	—
The attachment mode changes the selectivity only at the margin	yes	—	yes	yes	yes	yes
Non-basic silanes raise the elimination barrier; δPA ranks the series	—	—	—	yes	—	—

## Data Availability

The original contributions presented in this study are included in the article. The raw data supporting the conclusions of this article are available upon request from the corresponding author.

## Supplementary Materials

The following supporting information can be downloaded at: https://www.mdpi.com/article/10.3390/nano16201316/s1, Table S1: Conformer selection windows; Table S2: Transition-state searches and conformational sampling; Table S3: Thermal-neutron absorption estimate: inputs and results; Table S4: Branching under six entropy treatments; Table S5: Electronic barrier difference $\Delta \Delta E^{‡}$ in every parameterized continuum; Table S6: PBE0-D3(BJ)/def2-SVP single points at the GFN2-xTB saddle points; Table S7: Transition states re-optimized at PBE0-D3(BJ)/def2-SVP/IEF-PCM(*ε* = 3.4); Table S8: Deuterium kinetic isotope effects, β-deuterated PVC model, 453 K; Table S9: Silane series, descriptor and verified elimination barriers; Table S10: Interface models; Table S11: Downhill relaxations from the DFT saddle points.

## Abbreviations

The following abbreviations are used in this manuscript:

ALPB	analytical linearized Poisson–Boltzmann solvation model
APTES	3-aminopropyltriethoxysilane
AS	3-aminopropylsilanetriol (amine model)
B3LYP	Becke three-parameter Lee–Yang–Parr hybrid functional
BDE	bond dissociation energy
BFGS	Broyden–Fletcher–Goldfarb–Shanno
BS	B–O–Si bridged grafted silane (amine model)
D3(0)	Grimme D3 dispersion correction with zero damping
D3(BJ)	Grimme D3 dispersion correction with Becke–Johnson damping
DFT	density functional theory
E1cB	unimolecular elimination through the conjugate base
E2	bimolecular elimination
ESR	electron spin resonance
ETKDG	experimental-torsion knowledge distance geometry
GFN1-xTB, GFN2-xTB	geometries, frequencies and noncovalent interactions extended tight-binding methods, versions 1 and 2
GPTMS	3-glycidoxypropyltrimethoxysilane
HDPE	high-density polyethylene
HOMO	highest occupied molecular orbital
IEF-PCM	integral-equation-formalism polarizable continuum model
LDPE	low-density polyethylene
LUMO	lowest unoccupied molecular orbital
M06-2X	Minnesota 2006 hybrid meta-generalized-gradient-approximation functional with doubled nonlocal exchange
MACE-MP-0	multi-atomic cluster expansion foundation potential trained on Materials Project data
MAE	mean absolute error
MMFF	Merck molecular force field
MPS	3-methacryloxypropyltrimethoxysilane
NIST	National Institute of Standards and Technology
NMR	nuclear magnetic resonance
PA	*n*-propylamine (amine model)
PBE0	Perdew–Burke–Ernzerhof hybrid functional
PVC	poly(vinyl chloride)
RMSD	root-mean-square deviation
RRHO	rigid-rotor harmonic-oscillator
SMILES	simplified molecular-input line-entry system
$S_{N}2$	bimolecular nucleophilic substitution
SVP, TZVP	split-valence and triple-zeta valence basis sets with polarization functions (def2 family)
TS	transition state
UFF	universal force field
UV	ultraviolet
XPS	X-ray photoelectron spectroscopy
