# Supplementary material for: Aminosilane Coupling Agents at the Boron Carbide/Poly(Vinyl Chloride) Interface: Substitution, Minor Dehydrochlorination and C–Cl Bond Weakening
Source: Nanomaterials (Basel). 2026 Oct 18;16(20):1316. doi: 10.3390/nano16201316 (PMC13707875; doi:10.3390/nano16201316)
Supplement: Supplementary file 1 [file nanomaterials-16-01316-s001.zip › nanomaterials-4572336-supplementary.pdf]

Supplementary Materials

Aminosilane Coupling Agents at the Boron Carbide/Poly(vinyl chloride) Interface: Substitution, Minor Dehydrochlorination and C–Cl Bond Weakening

Yetkin Senemoğlu

All values are computed in this work unless a source is given. Every table is generated programmatically from the machine-readable result files of the calculations. PA = *n*-propylamine, AS = 3-aminopropylsilanetriol, BS = B–O–Si bridged grafted silane in its monodentate (T^1), bidentate (T^2) and tridentate (T^3) forms. Energies in kJ mol⁻¹ unless stated otherwise.

Table S1. Conformer selection windows.

ETKDGv3 embedding (up to 150 conformers, RMSD pruning 0.6 Å, seed 20260831), force-field pre-optimization, ten lowest kept; for the hexamers and the tetramer two further conformers were refined as a check (Table 3 of the main text). Force-field windows in kJ mol^{-1} relative to the force-field minimum; the GFN2-xTB spread is the GFN2-xTB energy range of the refined set, in kJ mol^{-1}. The embedding counts are reproduced with the final settings; for 4-chloro-2-pentene these return a single conformer, whereas the refined set of Table 3 holds three conformers from the original embedding run, whose GFN2-xTB spread is the one given.

Species	Force field	Embedded	Kept	Kept window	Discarded window	GFN2-xTB spread
APTES (parent)	MMFF94s	30	10	0–6.0	6.0–24.6	15.4
APTES, monohydrolyzed	MMFF94s	39	10	0–13.9	14.4–27.2	18.2
APTES, dihydrolyzed	MMFF94s	27	10	0–22.0	22.4–33.0	21.4
3-Aminopropylsilanetriol	MMFF94s	7	7	0–31.4	none	16.5
B–O–Si bridged silane, T^1	UFF	62	10	0–1.9	2.3–15.5	24.5
PVC hexamer, *RSSRSR*	MMFF94s	150	10	0–17.6	19.0–83.3	16.7
PVC hexamer, *RRSSRS*	MMFF94s	148	10	0–25.9	26.2–110.5	17.7
PVC hexamer, *RSRSRS*	MMFF94s	148	10	0–9.8	11.1–84.3	9.7
PVC oligomer, n = 2, *RS*	MMFF94s	6	6	0–21.7	none	15.4
PVC tetramer, *RSSR*	MMFF94s	111	10	0–18.5	18.5–70.4	10.7
PVC dyad (2,4-dichloropentane)	MMFF94s	6	6	0–21.7	none	15.4
Allylic chloride (4-chloro-2-pentene)	MMFF94s	1	1	0–0.0	none	5.0
Water	MMFF94s	1	1	0–0.0	none	0.0
Ethanol	MMFF94s	1	1	0–0.0	none	0.0
Hydrogen chloride	UFF	1	1	0–0.0	none	0.0
Boric acid	UFF	1	1	0–0.0	none	0.0
n-propylamine	MMFF94s	2	2	0–2.3	none	1.7
Methanol	MMFF94s	1	1	0–0.0	none	0.0

Table S2. Transition-state searches and conformational sampling.

Original searches from the relaxed-scan maxima (GFN2-xTB and GFN1-xTB), and the conformational sampling of Section 2.4 (GFN2-xTB): starting structures, converged searches, structures passing the acceptance test and distinct transition-state conformers after merging duplicates. The rows include the chain-length and scan-schedule tests of Section 3.8 and the cluster models of Section 3.7. $\nu^{\ddagger}$ is the imaginary frequency; the overlap is with the seeding direction; the RMSD is from the scan maximum. Strategy is the search that produced the listed structure (Section 2.4): Cartesian coordinates seeded with the intended reaction coordinate, or one of the unseeded retries in internal coordinates or with an exact initial Hessian, whose saddle points were accepted only if their imaginary mode overlaps the intended reaction coordinate.

Reaction	Method	Optimizer runs	Strategy	N(imag)	$\nu^{\ddagger}$ / cm^{-1}	Overlap	RMSD / Å	Outcome
PA, PVC dyad, E2	GFN2-xTB	3	internal, unseeded	1	−1188.7	0.70	0.14	accepted
PA, PVC dyad, S_N2	GFN2-xTB	2	internal, unseeded	1	−521.5	0.43	0.15	accepted
AS, PVC dyad, E2	GFN2-xTB	2	internal, unseeded	1	−1177.3	0.66	1.35	accepted
AS, PVC dyad, S_N2	GFN2-xTB	2	internal, unseeded	1	−533.9	0.39	0.99	accepted
BS (T^1), PVC dyad, E2	GFN2-xTB	4	internal, unseeded	1	−1250.7	0.70	0.18	accepted
BS (T^1), PVC dyad, S_N2	GFN2-xTB	5	internal, unseeded	1	−537.3	0.42	0.55	accepted
BS (T^2), PVC dyad, E2	GFN2-xTB	5	Cartesian, exact Hessian	1	−1175.5	0.69	0.25	accepted
BS (T^2), PVC dyad, S_N2	GFN2-xTB	2	internal, unseeded	1	−518.4	0.43	0.23	accepted
BS (T^3), PVC dyad, E2	GFN2-xTB	5	Cartesian, exact Hessian	1	−1206.7	0.61	1.00	accepted
BS (T^3), PVC dyad, S_N2	GFN2-xTB	5	Cartesian, exact Hessian	1	−533.1	0.34	1.53	accepted
BS (T^1), PVC dyad, chain-length pair, E2	GFN2-xTB	6	internal, unseeded	2	−1235.8	0.70	0.17	accepted; soft second mode (−11.5 cm^{-1})
BS (T^1), PVC dyad, chain-length pair, S_N2	GFN2-xTB	5	Cartesian, exact Hessian	2	−539.4	0.42	0.14	accepted; soft second mode (−44.9 cm^{-1})
BS (T^1), PVC tetramer, E2	GFN2-xTB	5	Cartesian, exact Hessian	1	−1022.8	0.70	0.69	accepted
BS (T^1), PVC tetramer, S_N2	GFN2-xTB	2	internal, unseeded	1	−535.3	0.43	0.43	accepted
BS (T^1), PVC dyad, base-lagging scan, E2	GFN2-xTB	5	Cartesian, exact Hessian	1	−1262.2	0.69	0.15	accepted
BS (T^1), PVC dyad, base-lagging scan, S_N2	GFN2-xTB	5	Cartesian, exact Hessian	3	−539.6	0.42	0.19	rejected (3 imaginary modes)
BS (T^1) on a diborate, PVC dyad, E2	GFN2-xTB	1	Cartesian, seeded	1	−1218.3	0.70	0.83	accepted
BS (T^1) on a diborate, PVC dyad, S_N2	GFN2-xTB	6	Cartesian, seeded	2	−501.7	0.39	0.79	accepted; soft second mode (−15.4 cm^{-1})
BS (T^1) on a boroxol ring, PVC dyad, E2	GFN2-xTB	1	Cartesian, seeded	1	−1202.1	0.39	1.57	accepted

Reaction	Method	Optimizer runs	Strategy	*N*(imag)	$\nu^{\ddagger}$ / cm⁻¹	Overlap	RMSD / Å	Outcome
BS (T^1) on a boroxol ring, PVC dyad, S_N2	GFN2-xTB	1	Cartesian, seeded	1	−179.5	0.26	0.21	accepted
PA, allylic chloride, E2	GFN2-xTB	2	internal, unseeded	1	−1036.4	0.67	0.46	accepted
PA, allylic chloride, S_N2	GFN2-xTB	2	internal, unseeded	1	−417.0	0.47	0.10	accepted
AS, allylic chloride, E2	GFN2-xTB	4	internal, unseeded	1	−962.0	0.67	0.08	accepted
AS, allylic chloride, S_N2	GFN2-xTB	5	internal, unseeded	1	−410.2	0.45	1.37	accepted
BS (T^1), allylic chloride, E2	GFN2-xTB	2	internal, unseeded	1	−1049.1	0.68	0.35	accepted
BS (T^1), allylic chloride, S_N2	GFN2-xTB	5	Cartesian, exact Hessian	1	−419.9	0.48	0.09	accepted
PA, PVC dyad, E2	GFN1-xTB	6	internal, unseeded	2	−709.2	0.72	0.20	accepted; soft second mode (−27.1 cm⁻¹)
PA, PVC dyad, S_N2	GFN1-xTB	2	internal, unseeded	1	−634.0	0.48	0.15	accepted
AS, PVC dyad, E2	GFN1-xTB	2	internal, unseeded	1	−767.7	0.64	1.40	accepted
AS, PVC dyad, S_N2	GFN1-xTB	2	internal, unseeded	1	−646.1	0.46	1.23	accepted
BS (T^1), PVC dyad, E2	GFN1-xTB	6	Cartesian, seeded	2	−769.8	0.73	0.33	accepted; soft second mode (−22.0 cm⁻¹)
BS (T^1), PVC dyad, S_N2	GFN1-xTB	2	internal, unseeded	1	−639.6	0.47	0.39	accepted
BS (T^2), PVC dyad, E2	GFN1-xTB	2	internal, unseeded	1	−779.8	0.72	0.48	accepted
BS (T^2), PVC dyad, S_N2	GFN1-xTB	2	internal, unseeded	1	−631.1	0.47	0.55	accepted
BS (T^3), PVC dyad, E2	GFN1-xTB	2	internal, unseeded	1	−824.8	0.70	1.22	accepted
BS (T^3), PVC dyad, S_N2	GFN1-xTB	2	internal, unseeded	1	−633.1	0.35	1.74	accepted
PA, allylic chloride, E2	GFN1-xTB	2	internal, unseeded	1	−327.7	0.54	0.11	accepted
PA, allylic chloride, S_N2	GFN1-xTB	2	internal, unseeded	1	−537.7	0.50	0.15	accepted
AS, allylic chloride, E2	GFN1-xTB	2	internal, unseeded	1	−389.0	0.53	0.67	accepted
AS, allylic chloride, S_N2	GFN1-xTB	2	internal, unseeded	1	−515.9	0.49	1.53	accepted
BS (T^1), allylic chloride, E2	GFN1-xTB	6	internal, exact Hessian	1	−367.9	0.50	3.00	accepted
BS (T^1), allylic chloride, S_N2	GFN1-xTB	2	internal, unseeded	1	−550.7	0.42	1.65	accepted

Conformational sampling (GFN2-xTB/ALPB). The last column is the energy range of the distinct transition-state conformers, kJ mol^{-1}.

Transition state	Starts	Converged	Accepted	Distinct	Energy range / kJ mol^{-1}
PA, PVC dyad, E2	11	10	10	9	3.8
PA, PVC dyad, S_N2	11	10	10	10	17.7
AS, PVC dyad, E2	11	10	6	7	10.2
AS, PVC dyad, S_N2	11	10	9	10	14.5
BS (T^1), PVC dyad, E2	11	10	5	6	20.0
BS (T^1), PVC dyad, S_N2	11	10	8	9	12.6
BS (T^2), PVC dyad, E2	11	10	4	5	6.2
BS (T^2), PVC dyad, S_N2	11	10	7	7	35.7
BS (T^3), PVC dyad, E2	11	10	5	6	16.6
BS (T^3), PVC dyad, S_N2	11	10	10	11	34.1
PA, allylic chloride, E2	11	10	8	9	3.3
PA, allylic chloride, S_N2	11	10	10	11	9.4
AS, allylic chloride, E2	11	10	6	7	11.6
AS, allylic chloride, S_N2	11	10	9	10	7.2
BS (T^1), allylic chloride, E2	11	10	7	8	25.6
BS (T^1), allylic chloride, S_N2	11	10	10	11	26.4

Table S3. Thermal-neutron absorption estimate: inputs and results.

Microscopic cross sections at 2200 m s⁻¹ from Sears (*Neutron News* 1992, *3*, 26–37): absorption 0.3326 b (H), 0.0035 b (C), 33.5 b (Cl), 767 b (natural B). Void-free inverse rule of mixtures; infinite homogeneous medium; no transport, moderation, secondary gamma production, particle self-shielding or porosity. w is the mass fraction of B_4C, ρ the density, N(H) the number density of hydrogen atoms, Σ_a the macroscopic absorption cross section and λ_a the absorption mean free path; the last four columns give the contribution of each element to Σ_a, and a dash (—) marks an element absent from the matrix.

Matrix	$w(B_4C)$ / %	ρ / g cm⁻³	N(H) / cm⁻³	Σ_a / cm⁻¹	λ_a / cm	Σ_a(H) / cm⁻¹	Σ_a(C) / cm⁻¹	Σ_a(Cl) / cm⁻¹	Σ_a(B) / cm⁻¹
HDPE	10.0	1.013	7.829×10^{22}	3.414	0.293	0.02604	0.00014	—	3.38762
PVC	6.2	1.439	3.905×10^{22}	3.414	0.293	0.01299	0.00009	0.43603	2.96464
PVC	0.0	1.400	4.047×10^{22}	0.466	2.148	0.01346	0.00009	0.45193	0.00000
HDPE	0.0	0.950	8.157×10^{22}	0.027	36.666	0.02713	0.00014	—	0.00000
LDPE	0.0	0.920	7.900×10^{22}	0.026	37.861	0.02627	0.00014	—	0.00000

Table S4. Branching under six entropy treatments.

Percentage of reactive encounters entering elimination at 453 K (Wigner tunneling included), single transition-state pair per channel, $3N - 7$ modes each, both Hamiltonians with ALPB chloroform. The floors are in cm⁻¹. The conformer-summed values are given in Table 4 of the main text, and the electronic term in every continuum in Table S5.

(a) GFN2-xTB

Pair	quasi-RRHO	floor 0	floor 25	floor 50	floor 100	floor 150	thermal term (quasi-RRHO) / kJ mol⁻¹
AS, allylic chloride	83.6	99.0	97.1	92.6	72.8	60.4	17.5
AS, PVC dyad	96.9	97.6	98.6	98.8	97.9	97.8	19.6
BS (T^2), PVC dyad	97.5	99.0	99.2	99.1	98.2	97.0	19.9
BS (T^3), PVC dyad	94.7	98.2	97.7	97.9	94.8	92.8	20.7
BS (T^1), allylic chloride	89.0	98.2	98.2	96.8	89.3	83.6	18.8
BS (T^1), PVC dyad	99.9	100.0	100.0	100.0	99.9	99.9	21.2
BS (T^1), PVC dyad, chain-length pair	99.9	99.8	99.9	100.0	99.9	99.9	15.5
BS (T^1), PVC tetramer	97.8	97.8	99.3	99.5	98.9	98.8	14.3
PA, allylic chloride	75.0	98.8	95.8	84.4	55.3	38.3	21.7
PA, PVC dyad	99.1	99.9	99.8	99.6	99.0	98.2	23.0

(b) GFN1-xTB

Pair	quasi-RRHO	floor 0	floor 25	floor 50	floor 100	floor 150	thermal term (quasi-RRHO) / kJ mol⁻¹
AS, allylic chloride	76.5	95.7	90.9	85.2	65.6	58.1	13.0
AS, PVC dyad	97.2	98.0	99.0	99.3	98.6	98.0	12.7
BS (T^2), PVC dyad	97.6	98.4	99.1	99.1	98.7	98.1	14.5
BS (T^3), PVC dyad	94.9	95.2	98.2	98.4	97.6	96.4	13.1
BS (T^1), allylic chloride	87.1	93.5	90.3	92.3	86.5	83.8	8.1
BS (T^1), PVC dyad	99.7	99.9	99.9	99.9	99.6	99.5	18.0
PA, allylic chloride	67.3	91.8	84.6	67.8	56.8	44.5	14.2
PA, PVC dyad	95.7	98.1	98.5	98.6	97.2	95.4	15.9

Table S5. Electronic barrier difference $\Delta\Delta E^{\ddagger}$ in every parameterized continuum.

Values in kJ mol^{-1} from GFN2-xTB and GFN1-xTB single points at the chloroform geometries; positive favors elimination. The relative permittivity ε of each ALPB solvent is given in parentheses. The band used in the main text is the six solvents with $2.9 \le \varepsilon \le 8.9$.

(a) GFN2-xTB

Pair	vacuum	toluene (2.4)	carbon disulfide (2.6)	furan (2.9)	diethyl ether (4.3)	chloroform (4.7)	ethyl acetate (6.0)	tetrahydrofuran (7.4)	dichloromethane (8.9)	acetone (20.5)	acetonitrile (36.6)	dimethyl sulfoxide (46.7)	water (78.4)
AS, allylic chloride	−103.2	−42.1	−44.6	−28.4	−29.2	−12.4	−27.6	−24.3	−21.7	−24.5	−18.4	−15.9	14.1
AS, PVC dyad	−67.8	−28.8	−34.1	−17.6	−20.1	−8.0	−16.5	−16.8	−15.7	−18.6	−14.2	−13.6	13.6
BS (T^2), PVC dyad	−62.4	−25.8	−29.2	−17.0	−19.3	−7.5	−15.1	−16.2	−13.5	−16.2	−13.6	−12.4	7.3
BS (T^3), PVC dyad	−57.6	−26.7	−28.1	−17.7	−18.7	−11.2	−16.2	−17.2	−18.1	−19.5	−17.5	−15.5	2.3
BS (T^1), allylic chloride	−104.2	−42.7	−46.9	−29.5	−31.2	−12.1	−28.5	−25.5	−22.3	−25.8	−19.9	−15.3	12.7
BS (T^1), PVC dyad	−52.5	−16.3	−19.6	−6.8	−7.8	2.8	−5.0	−4.2	−4.2	−6.4	−3.8	−1.0	19.9
BS (T^1), PVC dyad, chain-length pair	−43.6	−9.2	−13.2	−0.7	−4.1	7.7	0.6	−1.2	1.4	−2.9	−0.2	1.5	21.8
BS (T^1), PVC tetramer	−39.2	−12.7	−19.3	−5.9	−7.9	−0.9	−7.7	−5.7	−6.6	−8.2	−6.3	−3.6	16.0
PA, allylic chloride	−121.1	−51.1	−56.8	−37.6	−40.8	−18.7	−36.3	−34.7	−28.7	−33.5	−26.2	−24.1	7.4
PA, PVC dyad	−61.4	−23.7	−28.6	−15.5	−17.3	−6.4	−14.2	−13.6	−12.1	−14.4	−11.6	−9.2	11.5

(b) GFN1-xTB

Pair	vacuum	toluene (2.4)	carbon disulfide (2.6)	furan (2.9)	diethyl ether (4.3)	chloroform (4.7)	ethyl acetate (6.0)	tetrahydrofuran (7.4)	dichloromethane (8.9)	acetone (20.5)	acetonitrile (36.6)	dimethyl sulfoxide (46.7)	water (78.4)
AS, allylic chloride	−40.6	−20.6	−17.1	−11.5	−16.4	−8.4	−8.8	−9.1	−7.4	−12.4	−3.7	−3.6	−9.5
AS, PVC dyad	−31.9	−12.4	−11.9	−3.1	−7.0	0.5	0.9	0.3	2.4	−1.7	5.6	6.0	3.5
BS (T^2), PVC dyad	−39.7	−15.7	−18.8	−5.8	−11.6	−0.8	−0.6	−1.1	1.8	−4.4	5.5	6.5	1.0
BS (T^3), PVC dyad	−25.4	−12.1	−16.1	−4.7	2.1	−2.4	0.5	3.2	−1.4	8.5	4.8	7.3	6.4
BS (T^1), allylic chloride	−29.8	−12.3	−11.3	−3.3	−3.7	−0.7	−2.4	−3.6	−0.7	0.8	1.1	0.9	2.4
BS (T^1), PVC dyad	−24.4	−7.1	−6.2	1.1	−3.9	3.3	3.5	2.1	4.8	−0.4	7.8	7.7	3.9
PA, allylic chloride	−55.5	−27.1	−27.0	−15.5	−18.4	−11.2	−11.4	−10.0	−9.9	−11.7	−5.2	−3.3	−11.5
PA, PVC dyad	−40.8	−18.3	−22.3	−8.8	−11.0	−4.3	−3.8	−3.1	−1.8	−4.2	1.9	3.8	−0.7

Table S6. PBE0-D3(BJ)/def2-SVP single points at the GFN2-xTB saddle points.

$\Delta\Delta E^{\ddagger} = E(\mathrm{TS,\ S_N2}) - E(\mathrm{TS,\ E2})$, kJ mol⁻¹; negative values favor substitution. A dash (—) marks values that were not computed.

Pair	gas phase / kJ mol⁻¹	IEF-PCM, ε = 3.4 / kJ mol⁻¹	IEF-PCM, ε = 7.4 / kJ mol⁻¹
BS (T^1), PVC dyad	−54.4	−29.9	−20.9
BS (T^1) on a diborate, PVC dyad	−3.3	5.4	7.6
PA, PVC dyad	−56.4	−33.1	−24.7
AS, PVC dyad	−81.6	−49.2	—
BS (T^2), PVC dyad	−62.5	−38.9	—
BS (T^3), PVC dyad	−60.4	−42.5	—
PA, allylic chloride	−112.4	−55.5	—
AS, allylic chloride	−91.0	−41.7	—
BS (T^1), allylic chloride	−105.2	−54.9	—
BS (T^1) on a boroxol ring, PVC dyad	59.4	47.5	—

Table S7. Transition states re-optimized at PBE0-D3(BJ)/def2-SVP/IEF-PCM(ε = 3.4).

Verification on the continuum surface in the nine-dimensional subspace of Section 2.1: number of negative eigenvalues of the projected Hessian, the corresponding imaginary frequency $\nu^{\ddagger}$, and the overlap of its eigenvector with the GFN2-xTB reaction mode. The final maximum force is in eV Å⁻¹; RMSD from the GFN2-xTB saddle point in Å. The diborate substitution search did not converge; its structure, taken at the point of lowest residual force, is an approximate saddle point and not a converged transition state, and Section 3.7 of the main text uses it only qualitatively. Downhill relaxations from the four saddle points of the two principal pairs are given in Table S11.

Transition state	Maximum force / eV Å⁻¹	Negative eigenvalues	$\nu^{\ddagger}$ / cm⁻¹	Overlap with GFN2-xTB mode	RMSD / Å
PA, PVC dyad, E2	< 0.025	1	−128.2	0.467	0.307
PA, PVC dyad, S_N2	< 0.025	1	−426.3	0.997	0.054
BS (T^1), PVC dyad, E2	< 0.025	1	−124.5	0.401	0.629
BS (T^1), PVC dyad, S_N2	0.035	1	−443.5	0.991	0.248
BS (T^1) on a diborate, PVC dyad, E2	0.046	1	−109.6	0.421	0.164
BS (T^1) on a diborate, PVC dyad, S_N2	0.174	1	−404.8	0.968	0.656

Table S8. Deuterium kinetic isotope effects, β-deuterated PVC model, 453 K.

GFN2-xTB. H→D is the number of β-hydrogens replaced by deuterium; $\nu^{\ddagger}$ is the imaginary frequency of the protiated (H) and deuterated (D) transition state.

Pair	Channel	H→D	$\nu^{\ddagger}$(H) / cm⁻¹	$\nu^{\ddagger}$(D) / cm⁻¹	k_H/k_D	k_H/k_D with Wigner
PA, PVC dyad	E2	8	−1188.7	−898.4	2.96	3.52
PA, PVC dyad	S_N2	8	−521.5	−511.3	0.94	0.95
AS, PVC dyad	E2	8	−1177.3	−887.3	3.01	3.58
AS, PVC dyad	S_N2	8	−533.9	−524.1	0.91	0.91
BS (T^1), PVC dyad	E2	8	−1250.7	−943.2	2.90	3.50
BS (T^1), PVC dyad	S_N2	8	−537.3	−527.5	0.89	0.90

Table S9. Silane series, descriptor and verified elimination barriers.

GFN2-xTB/ALPB chloroform; δPA, $\Delta E^{\ddagger}$ and $\Delta G^{\ddagger}$ (quasi-RRHO, 453 K) in kJ mol⁻¹, $\nu^{\ddagger}$ in cm⁻¹. δPA is given for the lowest single conformer and for the conformer ensembles used in the main text; the shortest X–H···Y distance is the intramolecular hydrogen bond of the protonated form. A dash (—) marks quantities that are not defined, because glycidoxypropyl has no elimination saddle point.

Grafted silane	δPA, single conformer / kJ mol⁻¹	δPA, ensemble / kJ mol⁻¹	Shortest X–H···Y (protonated) / Å	Elimination TS	$\nu^{\ddagger}$ / cm⁻¹	$\Delta E^{\ddagger}$ / kJ mol⁻¹	$\Delta G^{\ddagger}$ / kJ mol⁻¹
Aminopropyl	0.0	0.0	1.81	yes	−1191	114.8	101.9
Acetamidopropyl	0.1	−59.7	1.69	yes	−991	153.7	136.8
N-methylaminopropyl	8.7	10.7	1.88	yes	−1146	112.2	98.0
N,*N*-dimethylaminopropyl	24.9	9.4	1.91	yes	−1279	109.4	99.0
Methacryloxypropyl	−105.4	−103.5	2.14	yes	−792	189.4	169.1
Glycidoxypropyl	−176.6	−131.2	1.46	no saddle point	—	—	—

Table S10. Interface models.

Association of *n*-propylamine with boron sites, kJ mol⁻¹, 1 mol L⁻¹ standard state; ΔG at the GFN2-xTB/ALPB level with quasi-RRHO thermochemistry, ΔE as the electronic association energy. The hydrogen-bonded boric acid complex retains one soft mode of −24 cm⁻¹, treated as a low-frequency vibration.

(a) Amine–boron association

Boron species	Complex	d(N–B) / Å	ΔG, 298 K / kJ mol⁻¹	ΔG, 453 K / kJ mol⁻¹	ΔE, GFN2-xTB / kJ mol⁻¹	ΔE, PBE0-D3(BJ) / kJ mol⁻¹
Boric acid, $B(OH)_3$	started as N→B adduct; relaxed to hydrogen-bonded	3.54	20.3	40.1	−19.2	−46.0
Boric acid, $B(OH)_3$	hydrogen-bonded	3.39	6.8	26.3	−31.9	−55.9
Boroxine, $B_3O_3(OH)_3$	dative N→B adduct	1.75	22.0	46.0	−28.3	−82.1
Boroxine, $B_3O_3(OH)_3$	hydrogen-bonded	3.27	4.1	26.1	−38.9	−62.1

Grafted-silane/PVC dyad on larger clusters and with explicit water, GFN2-xTB/ALPB: quasi-RRHO thermal term (kJ mol⁻¹) and percentage of elimination over the dielectric band (minimum, median, maximum). Both one-water transition states carry a soft second mode (−20 and −17 cm⁻¹). The diborate pair re-optimized at PBE0-D3(BJ)/def2-SVP/IEF-PCM favors substitution instead ($\Delta\Delta G^{\ddagger}$ = −31 kJ mol⁻¹; Table S7 and Section 3.7 of the main text), with an approximate substitution saddle point, so that this value indicates the sign of the preference and not its size. On the diborate, the only larger cluster re-optimized at the DFT level, the two levels of theory therefore disagree, and the values in this panel are semiempirical results only.

(b) Larger clusters and explicit water, monodentate graft

Model	Thermal term / kJ mol⁻¹	E2 / %, minimum	E2 / %, median	E2 / %, maximum
Diborate cluster (B–O–B bridge)	27.8	100	100	100
Boroxol-ring cluster (substitution TS marginal, $\nu^{\ddagger}$ = −180 cm⁻¹)	4.9	100	100	100
Single boron site, one H_2O at the leaving Cl	23.6	98	99	100

Table S11. Downhill relaxations from the DFT saddle points.

Each saddle point of the two principal pairs listed in Table S7 was displaced along the reaction mode of the mixed DFT/GFN2-xTB Hessian described in Section 2.1 of the main text, by 0.10 Å for the atom that moves most, in both directions, and relaxed at PBE0-D3(BJ)/def2-SVP/IEF-PCM(ε = 3.4) with a quasi-Newton optimizer with a maximum step of 0.15 Å, whose initial Hessian was that mixed Hessian with its eigenvalues taken by magnitude, until the bonds formed and broken had identified one side of the reaction and the maximum force had fallen below 0.5 eV Å^{-1}; the two substitution relaxations of the grafted silane (BS), which were slower, were instead stopped once the assignment had held for three consecutive steps, at the higher final forces listed. The product side of elimination is assigned when d(Cα–Cl) > 2.8 Å, $d(\mathrm{C\beta} - \mathrm{H_\beta})$ > 1.7 Å, $d(\mathrm{N} - \mathrm{H_\beta})$ < 1.12 Å and d(Cα–Cβ) < 1.40 Å, its reactant side when $d(\mathrm{C\beta} - \mathrm{H_\beta})$ < 1.15 Å, $d(\mathrm{N} - \mathrm{H_\beta})$ > 1.6 Å and d(Cα–Cl) < 2.0 Å; the product side of substitution when d(Cα–N) < 1.6 Å and d(Cα–Cl) > 2.8 Å, its reactant side when d(Cα–Cl) < 2.0 Å and d(Cα–N) > 2.7 Å. The relaxations establish connectivity and do not trace the minimum-energy path. Gradients is the number of DFT energy and gradient evaluations, including the displaced starting structure. ΔE is the electronic energy of the last structure relative to the saddle point, in kJ mol^{-1}; distances in Å; F_{max} is the final maximum atomic force in eV Å^{-1}. Cα carries the reacting chlorine, Cβ the transferred hydrogen $\mathrm{H_\beta}$. The +/− sign of the displacement follows the eigenvector and has no chemical meaning.

Saddle point	Direction	Gradients	ΔE / kJ mol^{-1}	d(Cα–Cl) / Å	d(Cα–N) / Å	$d(\mathrm{N} - \mathrm{H_\beta})$ / Å	$d(\mathrm{C\beta} - \mathrm{H_\beta})$ / Å	d(Cα–Cβ) / Å	F_{max} / eV Å^{-1}	End point
PA, PVC dyad, E2	+	16	−130.4	1.82	3.88	2.29	1.10	1.51	0.371	reactant complex
PA, PVC dyad, E2	−	7	−6.7	2.85	3.27	1.07	1.90	1.35	0.369	alkene + alkylammonium chloride
PA, PVC dyad, $\mathrm{S_N2}$	+	12	−78.1	1.87	2.96	2.84	1.11	1.52	0.496	reactant complex
PA, PVC dyad, $\mathrm{S_N2}$	−	16	−86.9	4.13	1.56	2.60	1.10	1.53	0.449	alkylammonium chloride
BS ($\mathrm{T^1}$), PVC dyad, E2	+	9	−131.3	1.81	3.77	2.20	1.11	1.51	0.427	reactant complex
BS ($\mathrm{T^1}$), PVC dyad, E2	−	7	−7.8	2.88	3.19	1.06	2.04	1.35	0.356	alkene + alkylammonium chloride
BS ($\mathrm{T^1}$), PVC dyad, $\mathrm{S_N2}$	+	10	−89.2	3.49	1.59	2.58	1.10	1.53	1.525	alkylammonium chloride
BS ($\mathrm{T^1}$), PVC dyad, $\mathrm{S_N2}$	−	11	−63.3	1.87	2.77	2.98	1.11	1.52	0.837	reactant complex
